# Musical Creativity and Depth of Implicit Knowledge: Spectral and Temporal Individualities in Improvisation

**DOI:** 10.3389/fncom.2018.00089

**Published:** 2018-11-13

**Authors:** Tatsuya Daikoku

**Affiliations:** Department of Neuropsychology, Max Planck Institute for Human Cognitive and Brain Sciences, Leipzig, Germany

**Keywords:** Implicit learning, statistical learning, n-gram, Markov model, entropy, characteristics, uncertainty, hierarchy

## Abstract

It has been suggested that musical creativity is mainly formed by implicit knowledge. However, the types of spectro-temporal features and depth of the implicit knowledge forming individualities of improvisation are unknown. This study, using various-order Markov models on implicit statistical learning, investigated spectro-temporal statistics among musicians. The results suggested that lower-order models on implicit knowledge represented general characteristics shared among musicians, whereas higher-order models detected specific characteristics unique to each musician. Second, individuality may essentially be formed by pitch but not rhythm, whereas the rhythms may allow the individuality of pitches to strengthen. Third, time-course variation of musical creativity formed by implicit knowledge and uncertainty (i.e., entropy) may occur in a musician's lifetime. Individuality of improvisational creativity may be formed by deeper but not superficial implicit knowledge of pitches, and that the rhythms may allow the individuality of pitches to strengthen. Individualities of the creativity may shift over a musician's lifetime via experience and training.

## Introduction

### Implicit knowledge and creativity in brain

The brain models external phenomena as a hierarchy of statistical dynamical systems, which encode causal chain structure in the sensorium (Friston et al., 2006; Friston and Kiebel, 2009; Friston, 2010) to maintain low entropy and free energy in the brain (von Helmholtz, 1909), and predicts a future state based on the internalized stochastic model to minimize sensory reaction and optimize motor action regardless of consciousness (Friston, 2005). This prediction associates with the brain's implicit, domain-general, and innate system, called implicit learning or statistical learning (Reber, 1967; Saffran et al., 1996; Cleeremans et al., 1998; Perruchet and Pacton, 2006), in which our brain automatically calculates transitional probabilities (TPs) of sequential phenomena and grasps information dynamics. The terms implicit learning and statistical learning have been used interchangeably and are regarded as the same phenomenon (Perruchet and Pacton, 2006). Because of the implicitness of statistical learning and knowledge, humans are unaware of exactly what they learn (Daikoku et al., 2014). Nonetheless, neurophysiological and behavioral responses disclose implicit learning effects (Francois and Schön, 2011; François et al., 2013; Daikoku et al., 2015, 2016, 2017a,c,d; Koelsch et al., 2016; Yumoto and Daikoku, 2016, 2018; Daikoku and Yumoto, 2017). When the brain implicitly encodes TP distributions that are inherent in dynamical phenomena, several things are automatically expected, including a probable future state with a higher TP, facilitating optimisation of performance based on the encoded statistics despite being unable to describe the knowledge (Broadbent, 1977; Berry and Broadbent, 1984; Green and Hecht, 1992; Williams, 2005; Rebuschat and Williams, 2012), and inhibit neurophysiological response to predictable external stimuli for the efficiency and low entropy of neural processing based on predictive coding (Daikoku, 2018b). The implicit knowledge has been considered to contribute to many types of mental representation: the comprehension and production of complex structural information such as music and language (Rohrmeier and Rebuschat, 2012), intuitive decision-making (Berry and Dienes, 1993; Reber, 1993; Perkovic and Orquin, 2017), auditory-motor planning (Pearce et al., 2010a,b; Norgaard, 2014), and creativity (Wiggins, 2018) involved in musical composition (Pearce and Wiggins, 2012; Daikoku, 2018a) and musical improvisation (Norgaard, 2014). Additionally, compared to language (Chomsky, 1957; Jackendoff and Lerdahl, 2006), several studies suggest that musical representation including tonality is mainly formed by a tacit knowledge (Delie‘ge et al., 1996; Delie‘ge, 2001; Bigand and Poulin-Charronnat, 2006; Ettlinger et al., 2011; Koelsch, 2011; Huron, 2012). Thus, it is widely accepted that implicit knowledge causes a sense of intuition, spontaneous behavior, skill acquisition based on procedural learning, and is further closely tied to musical production such as intuitive creativity, composition, and playing.

Particularly in musical improvisation, musicians are forced to express intuitive creativity and immediately play their own music based on long-term training associated with procedural and implicit learning (Clark and Squire, 1998; Ullman, 2001; Paradis, 2004; De Jong, 2005; Ellis, 2009; Müller et al., 2016). Thus, compared to other types of musical composition in which a composer deliberates and refines a composition scheme for a long time based on musical theory, the performance of musical improvisation is intimately bound to implicit knowledge because of the necessity of intuitive decision-making (Berry and Dienes, 1993; Reber, 1993; Perkovic and Orquin, 2017) and auditory-motor planning based on procedural knowledge (Pearce et al., 2010a,b; Norgaard, 2014). This suggests that the stochastic distribution calculated from musical improvisation may represent the musicians' implicit and statistical knowledge and individual creativity in music that has been developed via implicit learning. Few studies have investigated the relationship between musical improvisation and implicit knowledge. Here, this study proposed the computational model of improvisational creativity based on the framework of implicit statistical learning.

### Computational model of musical creativity

The computational model is often used to understand general music acquisition (Cilibrasi et al., 2004; Backer and van Kranenburg, 2005; Albrecht and Huron, 2012; Ito, 2012; Prince and Schmuckler, 2012; Albrecht and Shanahan, 2013; London, 2013), entropy-based music prediction (Manzara et al., 1992; Ian et al., 1994; Reis, 1999; Pearce and Wiggins, 2006; Cox, 2010), implicit learning, and the metal representation of implicit knowledge (Dubnov, 2010; Wang, 2010; Rohrmeier and Rebuschat, 2012). Particularly, Competitive Chunker (Servan-Schreiber and Anderson, 1990), PARSER (Perruchet and Vinter, 1998), Information Dynamics of Music (IDyOM) (Pearce, 2005; Pearce and Wiggins, 2012), and n-gram models (Pearce and Wiggins, 2004) underpin the hypothesis that music is acquired by extracting and concatenating chunks, which is a main theory of implicit learning and statistical learning. Although experimental approaches are necessary for understanding the real-world brain's function in music acquisition, the modeling approaches partially outperform experimental results under conditions that are impossible to replicate in an experimental approach. For example, they can directly verify much of the real-world music and time-course variation over long time periods (Daikoku, 2018a). Most experimental approaches use the specific paradigms, which are ecologically unrealistic and focus on the specific type of short-term learning effects (e.g., chord perception, prediction, and timing). Additionally, some modeling approaches calculate statistics in music and device models, and also evaluate the validities of these models by neurophysiological and behavioral experiments and provide possibilities of novel tasks for neural and behavioral experiments (Potter et al., 2007; Pearce et al., 2010a,b; Pearce and Wiggins, 2012). A combination of the two approaches is better because each can complement the weak points of the other approach (Daikoku, 2018b).

The n-gram models, which correspond to various-order Markov model (Markov, 1971), calculate TPs of sequences by chopping them into short fragments (n-grams) up to a size of n, and are frequently used in both experimental and computational approaches (Pearce and Wiggins, 2004; Daikoku, 2018b). The online musical production, however, is not the mere chopping of one type of length of sequence, but it is a dynamical prediction to maintain an aesthetic melody with various length of sequence, temporal, and spectral features, and harmony that interact with each other (Lerdahl and Jackendoff, 1983; Hauser et al., 2002; Jackendoff and Lerdahl, 2006). That is, the musical production is not restricted to a single stream of events or a hierarchy but, rather, they interact with various hierarchical structures. Previous computational (Conklin and Witten, 1995; Pearce and Wiggins, 2012) and neural studies (Daikoku and Yumoto, 2017) expanded the n-gram method to modeling the interaction of parallel streams and enhanced the predictive power. However, the model that suffices to explain musical creativity cannot still be devised. Nonetheless, the *n*th-order Markov models could explain that the prediction continually occurs with each state of sequence and that the entropy in the brain (i.e., the average surprise of outcomes sampled from a probability distribution, Applebaum, 2008) gradually decreases by exposure to musical sequences. Thus, the TP distribution sampled from music based on *n*th-order Markov models may refer to the characteristics of a composer's superficial-to-deep implicit knowledge: a high-probability transition in music may be one that a composer is more likely to predict and choose based on the latest n states, compared to a low-probability transition. The notion has also been neurophysiologically demonstrated by our previous studies (Daikoku et al., 2017b). The model has also been applied to develop artificial intelligence that give computers learning and decision-making abilities similar to that of the human brain, such as an automatic composition system (Raphael and Stoddard, 2004; Eigenfeldt, 2010; Boenn et al., 2012) and natural language processing (Brent, 1999; Manning and Schütze, 1999). Thus, the Markov model is used in the interdisciplinary realms of neuroscience, behavioral science, engineering, and informatics.

### Temporal and spectral feature in musical creativity

Temporal and spectral features are important pieces of information for which to configure characteristics of each type of music (e.g., individuality, genre, and culture). Additionally, two types of information are not independent of each other, but rather they closely interact. Thus, the relationships between temporal (i.e., rhythm) and spectral (i.e., melody) structures are a large question to understand music creativity. Some researchers indicated that humans cannot learn temporal structure independent of spectral structure (Buchner and Steffens, 2001; Shin and Ivry, 2002; O'Reilly et al., 2008), whereas other researchers demonstrated temporal implicit learning independent of pitch information (Salidis, 2001; Ullén and Bengtsson, 2003; Karabanov and Ulle'n, 2008; Brandon et al., 2012) and vice versa (Daikoku et al., 2017d). Additionally, neurophysiological and psychological studies suggested that humans can learn relative rather than absolute temporal and spectral (Daikoku et al., 2014, 2015) patterns. Thus, the relationships between temporal and spectral features on musical creativity and implicit learning remains controversial. To the best of my knowledge, there are no integrated models that cover temporal and spectral features in musical creativity. The present study first provides the implicit-learning models that unify temporal and spectral features in musical improvisation. Additionally, this study investigated which information (spectral and temporal) and hierarchy (1st to 6th orders) represent the individualities of creativity. To comprehensively understand how musical creativity occurs in the human brain and how temporal and spectral features are integrated to constitute musical individuality, it is necessary to investigate the relationships between spectral and temporal statistics inherent in music via various-order hierarchical models.

### Study purpose

The present study aimed to investigate the statistical differences and interactions between the temporal and spectral structure in improvisation among musicians using various-order Markov models, and to examine which information (spectral and temporal) and hierarchy represent the individualities of musical creativity. The statistical characteristics of the *n*th-order TP distribution of the spectral (pitch) and temporal sequences (pitch length and rest) in improvisational music were investigated. It was hypothesized that there were general statistical characteristics shared among musicians and specific statistical characteristics that were unique to each musician in both spectral and temporal sequences. Additionally, it was hypothesized that the detectability of the characteristics depends on hierarchy. If so, the individuality may depend on the depth of implicit knowledge. Furthermore, the chronological time-course variations of the entropies (uncertainly) and the predictability of each tone sequence were examined. It was hypothesized that implicit knowledge in music gradually shifts over a composer's lifetime. The present study first provided the findings on which information (spectral and temporal) and hierarchy (1st to 6th orders) represent the individualities of musical creativity.

## Methods

### Music information extraction

The music played by William John Evans (Autumn Leaves from Portrait in Jazz, 1959; Israel from Explorations, February 1961; I Love You Porgy from Waltz for Debby, June 1961; Stella by Starlight from Conversations with Myself, 1963; Who Can I Turn To? from Bill Evans at Town Hall, 1966; Someday My Prince Will Come from the Montreux Jazz Festival, 1968; A Time for Love from Alone, 1969), Herbert Jeffrey Hancock (Cantaloupe Island from Empyrean Isles, 1964; Maiden Voyage from Flood, 1975; Someday My Prince Will Come from The Piano, 1978; Dolphin Dance from Herbie Hancock Trio'81, 1981; Thieves in the Temple from The New Standard, 1996; Cottontail from Gershwin's World, 1998; The Sorcerer from Directions in Music, 2001), and McCoy Tyner (Man from Tanganyika from Tender Moments, 1967; Folks from Echoes of a Friend, 1972; You Stepped Out of a Dream from Fly with the Wind, 1976; For Tomorrow from Inner Voice; 1977; The Habana Sun from The Legend of the Hour, 1981; Autumn Leaves from Revelations, 1988; Just in Time from Dimensions, 1984) were used in the present study. The highest pitches including the length were chosen based on the following definitions: the highest pitches that can be played at a given point in time, pitches with slurs that can be counted as one, and grace notes were excluded. In addition, the rests that were related to highest-pitch sequences were also extracted. This spectral and temporal information were divided into four types of sequences: (1) a pitch sequence without length and rest information (i.e., pitch sequence without rhythms); (2) a rhythm sequence without pitch information (i.e., rhythm sequence without pitches); (3) a pitch sequence with length and rest information (i.e., pitch sequence with rhythms); and (4) a rhythm sequence with pitch information (i.e., rhythm sequence with pitches).

### Stochastic calculation

#### Pitch sequence without rhythms

**Figure 1:**
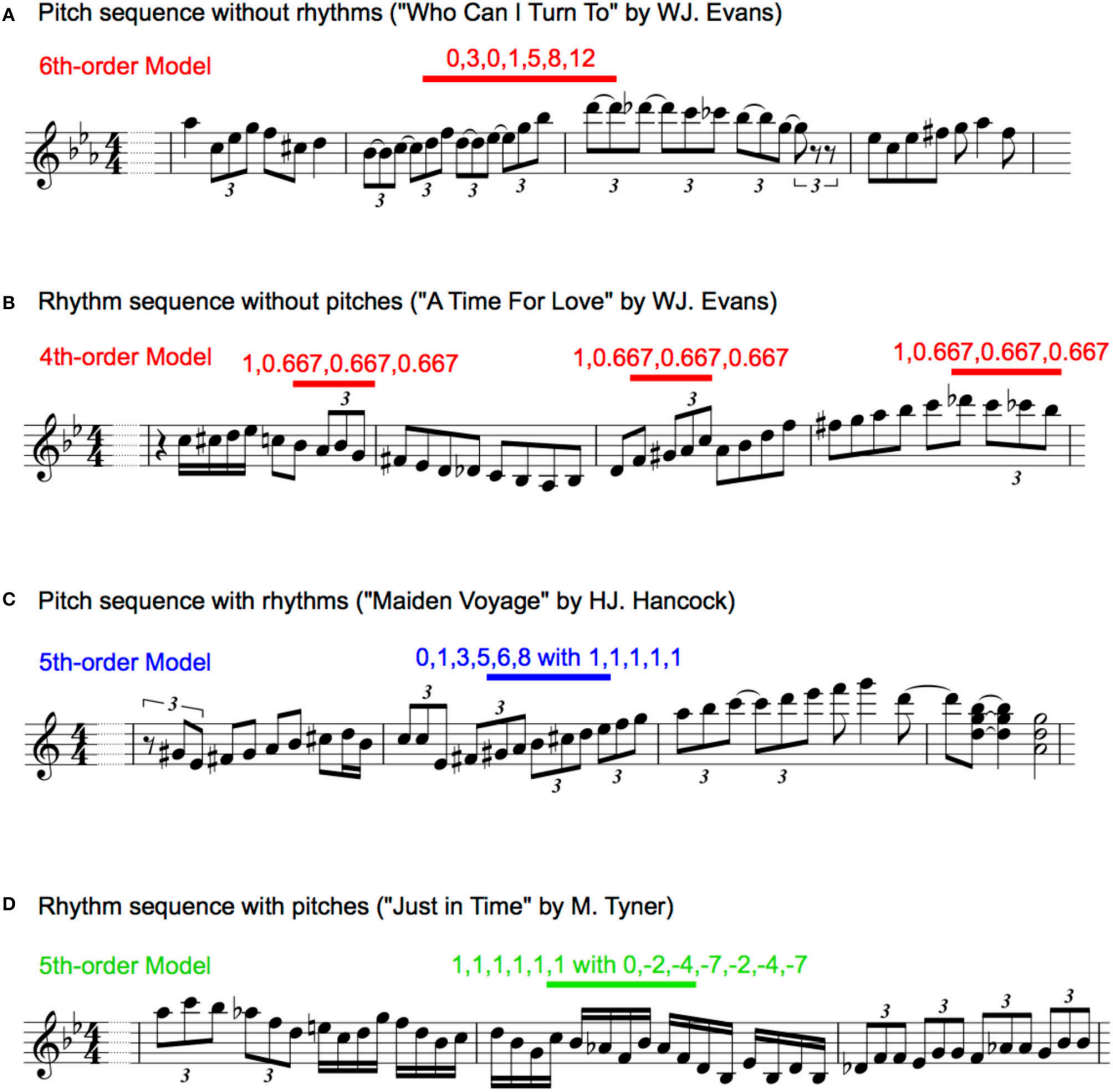
Representative phrases of transition patterns in pitch sequence without rhythms **(A)**, rhythm sequences without pitches **(B)**, pitch sequence with rhythms **(C)**, and rhythm sequences with pitches **(D)**. The musical information was extracted by listening music information recording media and originally written for the present study.

For each type of pitch sequence, all pitches were numbered so that the first pitch was 0 in each transition, and an increase or decrease in a semitone was 1 and −1 based on the first pitch, respectively. Representative examples were shown in Figure 1A. This revealed the relative pitch-interval patterns but not the absolute pitch patterns [30, 98]. This procedure was used to eliminate the effects of the change in key on transitional patterns. Interpretation of the key change depends on the musician, and it is difficult to define in an objective manner. Thus, the results in the present study may represent a variation in the statistics associated with relative pitch rather than absolute pitch. According to recent neurophysiological studies, human's implicit-learning system of auditory sequence capture relative rather than absolute transition patterns. In each piece of music for each musician, the TPs of the pitch sequences were calculated as a statistic based on multi-order Markov chains. The probability of a forthcoming pitch was statistically defined by the last pitch to six successive pitches (i.e., first- to six-order Markov chains). The *n*th-order Markov model is based on the conditional probability of an element e_n+1_, given the preceding *n* elements:

(1) $$\begin{array}{l} P\left(e_{n+1}|e_{n}\right)\;=\;\frac{P\left(e_{n+1}\cap e_{n}\right)}{P\left(e_{n}\right)} \end{array}$$

#### Rhythm sequence without pitches

The onset times of each note were used for analyses. Although note onsets ignore the length of notes and rests, this methodology can capture the most essential rhythmic features of the music [30,99]. To extract a temporal interval between adjacent notes, all onset times were subtracted from the onset of the preceding note. Then, for each type of rhythm sequence, the second to last temporal interval was divided by the first temporal interval. Representative examples are shown in Figure 1B. This revealed relative rhythm patterns but not absolute rhythm patterns; it is independent of the tempo of each piece of music. In each piece of music in each musician, the TPs of the rhythm sequences were calculated as a statistic based on multi-order Markov chains. The probability of a forthcoming temporal interval was statistically defined by the last temporal interval to six successive temporal intervals, respectively (i.e., first- to six-order Markov chains).

#### Pitch sequence with rhythms

The two methodologies of pitch and rhythm sequences were combined. For each type of sequence, all pitches were numbered so that the first pitch was 0 in each transition, and an increase or decrease in a semitone was 1 and −1 based on the first pitch, respectively. Additionally, for each type of pitch sequence, all onset times were subtracted from the onset of the preceding note, and the second to last temporal intervals were divided by the first temporal interval. The representative examples were shown in Figure 1C. For each piece of music for each musician, the TPs of the pitch sequences with rhythms were calculated as a statistic based on multi-order Markov chains. The probability of a forthcoming pitch with temporal information was statistically defined by the last pitch with temporal information to six successive pitches with temporal information, respectively (i.e., first- to six-order Markov chains). In the first-order hierarchical model of the pitch sequence with rhythms, a temporal interval was calculated as a ratio to the crotchet (i.e., quarter note), because only a temporal interval is included for each sequence and the note length cannot be calculated as a relative temporal interval. Thus, the patterns of pitch sequence (p) with rhythms (r) were represented as [p] with [r].

#### Rhythm sequence with pitches

The methodologies of sequence extraction were the same as those of the pitch sequence with rhythm (see Figure 1D), whereas the TPs of the rhythm, but not pitch, sequences were calculated as a statistic based on multi-order Markov chains. The probability of a forthcoming temporal interval with pitch was statistically defined by the last temporal interval with pitch to six successive temporal interval with pitch (i.e., first- to six-order Markov chains). Thus, the relative pattern of rhythm sequence (r) with pitches (p) were represented as [r] with [p].

### Statistical analysis

The TP distributions were analyzed by principal component analysis. The criteria of eigenvalue were set over 1. The first two components (i.e., the first and second highest cumulative contribution ratios) were adopted in the present study. Then, the information contents [*I(e*_*n*+1_*|e*_*n*_*)*] of TP were calculated based on information theory (Shannon, 1951). Furthermore, the *conditional entropy* [*H*(*AB*)] in n-order was calculated from information content:

(2) $$\begin{array}{l} I\left(e_{n+1}|e_{n}\right)=log_{2}\;\frac{1}{P\left(e_{n+1}|e_{n}\right)}\;\left(bit\right) \end{array}$$

(3) $$\begin{array}{l} H\left(B|A\right)={-\sum }_{i}\;\underset{j}{\sum }P\left(ai\right)P\left(bj|ai\right)\;log_{2}\;P\left(bj|ai\right)\;\left(bit\right) \end{array}$$

where *P(*bj|ai*)* is a conditional probability of sequence “*ai bj.”* The entropy were chronologically ordered based on the time courses in which music is played in each musician. The time-course variations of the entropies were analyzed by multiple regression analyses using the stepwise method. The criteria of the variance inflation factor (VIF) and condition index (CI) were set at VIF < 2 and CI < 20 to confirm that there was no multi collinearity (Cohen et al., 2003).

Furthermore, in each musician, seven pieces of music were averaged in each type of sequence. The transitional patterns with first to fifth highest TPs in each musician, which show higher predictabilities in each musician, were used in the regression analyses. The transitional patterns were chronologically ordered based on the time courses in which music is played in each musician. The time-course variations of the TPs were analyzed by multiple regression analyses using the stepwise method. The criteria of the variance inflation factor (VIF) and condition index (CI) were set at VIF < 2 and CI < 20 to confirm that there was no multi collinearity.

The logit transformation was applied to normalize the TPs. Then, using the transitional patterns with first to fifth highest TPs in each musician, the repeated-measure analysis of variances (ANOVAs) with a between-factor player (WJ. Evans vs. HJ. Hancock vs. M. Tyner) and a within-factor sequences for each hierarchy of Markov model were conducted. When we detected significant effects, Bonferroni-corrected *post-hoc* tests were conducted for further analysis. Statistical significance levels were set at *p* = 0.05 for all analyses.

## Results

### PCA

#### Pitch sequence without rhythms

**Figure 2:**
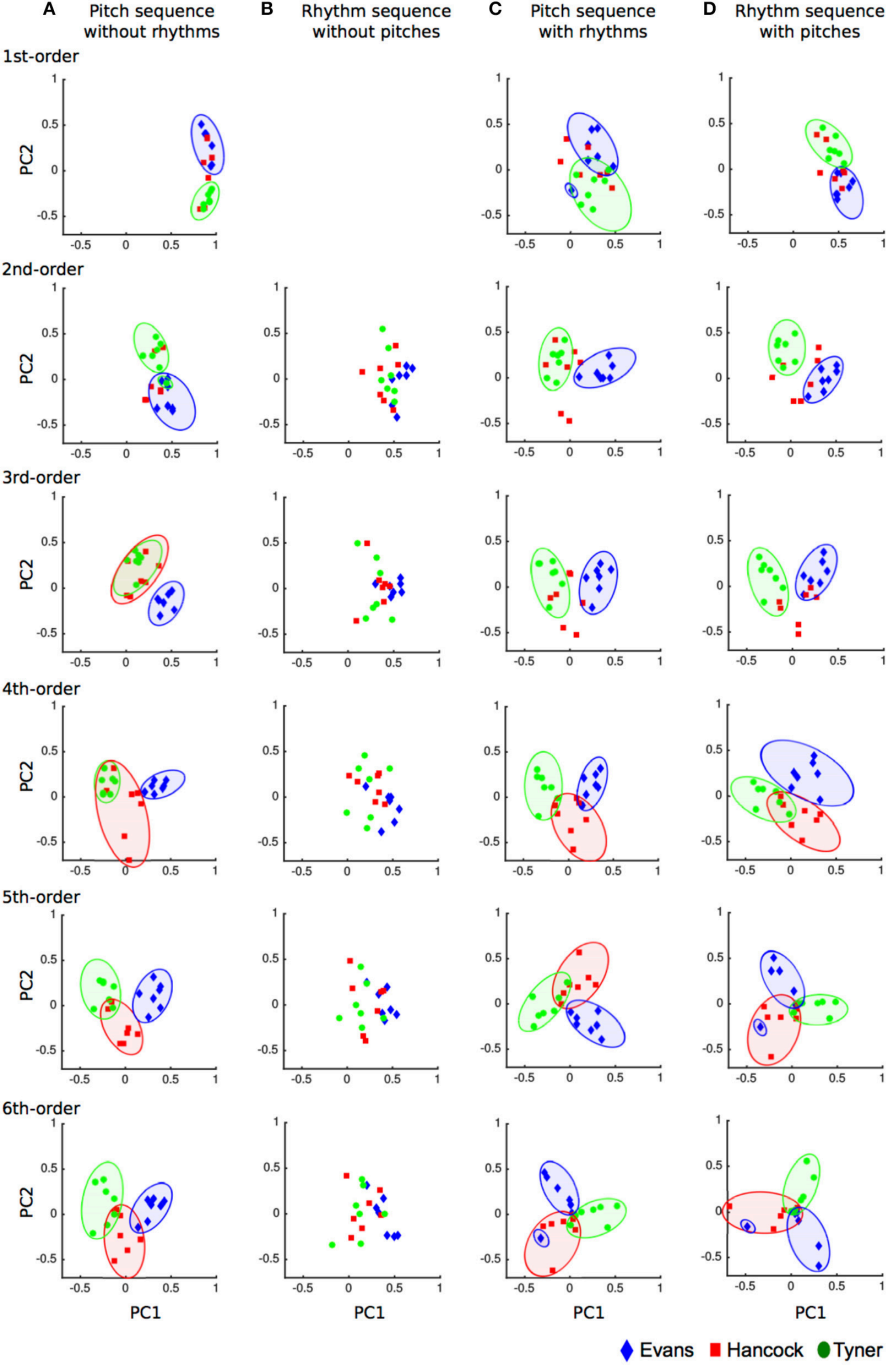
Principal component analysis scatter plots in pitch sequence without rhythms **(A)**, rhythm sequences without pitches **(B)**, pitch sequence with rhythms **(C)**, and rhythm sequence with pitches **(D)**. The horizontal and vertical axes represent principal component 1 and 2, respectively. The dots represent each piece of music.

The eigenvalue and percentages of variance, and the comulative variance and the eigenvectors for the principal components was shown in a Supplementary File. In the first-order hierarchical model (Figure 2A), the two components accounted for 91.445% of the total variance. All of the pieces of music loaded higher than.82 on component 1, suggesting that this explains the general component of jazz musical improvisation in three musicians. The eigenvectors of the pieces of music by W. J. Evans were higher than M. Tyner in component 2, suggesting that this explains a component of W. J. Evans or M. Tyner. The component of H. J. Hancock could not be detected. In the second-order hierarchical model, the two components accounted for 20.365% of the total variance. All of the pieces of music loaded higher than.18 on component 1, suggesting that this explains the general component of jazz musical improvisation in three musicians. In M. Tyner, the eigenvectors other than “The habana sun” were higher than W. J. Evans in component 2, suggesting that this explains a component of W. J. Evans or M. Tyner. The component of H. J. Hancock could not be detected. In the third-order hierarchical model, the two components accounted for 13.818% of the total variance. In H. J. Hancock and M. Tyner, the eigenvectors other than “Cotton tail” were lower than W. J. Evans in component 1, suggesting that this explains a component of W. J. Evans or a component combining H. J. Hancock and M. Tyner. No obvious difference among musicians could be detected in component 2. In the forth-, fifth-, and sixth-order hierarchical models, the two components accounted for 11.663, 10.968, and 10.586% of the total variance, respectively. The eigenvectors of the pieces of music by W. J. Evans were higher than H. J. Hancock and M. Tyner in component 1, suggesting that this explains a component of W. J. Evans or a component combining H. J. Hancock and M. Tyner. The eigenvectors of the pieces of music by H. J. Hancock were generally lower than W. J. Evans and M. Tyner in component 2, suggesting that this explains a weak component of H. J. Hancock or a component combining W. J. Evans and M. Tyner.

#### Rhythm sequence without pitches

In the first-order hierarchical model (Figure 2B), only one component, which accounted for 98.685% of the total variance, could be detected. The two components accounted for 91.445% of the total variance. All of the pieces of music loaded higher than.95 on the component, suggesting that this explains the general component of jazz musical improvisation in three musicians. In the second-, third-, forth, fifth-, and sixth-order hierarchical models, the two components accounted for 29.325, 20.985, 17.153, 14.780, and 13.376% of the total variance, respectively. No obvious difference among musicians could be detected in stochastic models of rhythms.

#### Pitch sequence with rhythms

In the first-order hierarchical models (Figure 2C), the two components accounted for 13.481% of the total variance. No obvious difference among musicians could be detected in component 1. In W. J. Evans, the eigenvectors other than “I love you porgy” were higher than M. Tyner in component 2, suggesting that this explains a component of W. J. Evans or M. Tyner. In the second-order hierarchical models, the two components accounted for 11.558% of the total variance. In W. J. Evans, the eigenvectors other than “I love you porgy” were higher than H. J. Hancock and M. Tyner in component 1, suggesting that this explains a component of W. J. Evans or a component combining H. J. Hancock and M. Tyner. No obvious difference among musicians could be detected in component 2. In the third-order hierarchical model, the two components accounted for 10.970% of the total variance. The eigenvectors of the pieces of music by W. J. Evans were higher than H. J. Hancock and M. Tyner in component 1, suggesting that this explains a component of W. J. Evans or a component combining H. J. Hancock and M. Tyner. No obvious difference among musicians could be detected in component 2. In the forth-order hierarchical model, the two components accounted for 10.774% of the total variance. In H. J. Hancock and M. Tyner, the eigenvectors other than “Dolphin dance” were lower than W. J. Evans in component 1, suggesting that this explains a component of W. J. Evans or a component combining H. J. Hancock and M. Tyner. The eigenvectors of the pieces of music by H. J. Hancock were generally lower than W. J. Evans and M. Tyner in component 2, suggesting that this explains a weak component of H. J. Hancock or a component combining W. J. Evans and M. Tyner. In the fifth-order hierarchical model, the two components accounted for 10.515% of the total variance. The eigenvectors of the pieces of music by W. J. Evans were higher than M. Tyner in component 1 and lower than H. J. Hancock in component 2, suggesting that these explain components of W. J. Evans, M. Tyner, and H. J. Hancock. In the sixth-order hierarchical model, the two components accounted for 10.344% of the total variance. In M. Tyner, the eigenvectors other than “For tomorrow” were higher than W. J. Evans and H. J. Hancock in component 1, suggesting that this explains a component of M. Tyner or a component combining W. J. Evans and H. J. Hancock. In W. J. Evans, the eigenvectors other than “Israel” were higher than H. J. Hancock in component 2, suggesting that these explain components of W. J. Evans or H. J. Hancock.

#### Rhythm sequence with pitches

In the first-order hierarchical model (Figure 2D), the two components accounted for 27.736% of the total variance. All of the pieces of music loaded higher than.25 on component 1, suggesting that this explains the general component of jazz musical improvisation in three musicians. The eigenvectors of the pieces of music by W. J. Evans were lower than M. Tyner in component 2, suggesting that this explains a component of W. J. Evans or M. Tyner. In the second-order hierarchical model, the two components accounted for 12.561% of the total variance. The eigenvectors of the pieces of music by W. J. Evans were higher than M. Tyner in component 1, suggesting that this explains a component of W. J. Evans or M. Tyner. No obvious difference among musicians could be detected in component 2. In the third- and forth-order hierarchical models, the two components accounted for 11.135 and 10.658% of the total variance, respectively. The eigenvectors of the pieces of music by W. J. Evans were higher than M. Tyner in component 1, suggesting that this explains a component of W. J. Evans or M. Tyner. In W. J. Evans, the eigenvectors other than “I love you porgy” in the third- and “Israel” in the forth-order hierarchical models were higher than H. J. Hancock in component 2, suggesting that this explains a component of W. J. Evans or H. J. Hancock. In the fifth-order hierarchical model, the two components accounted for 10.386% of the total variance. In M. Tyner, the eigenvectors other than “Autumn leaves” were higher than W. J. Evans in component 1, suggesting that this explains a component of W. J. Evans or M. Tyner. Tyner. The eigenvectors of the pieces of music by H. J. Hancock were generally lower than W. J. Evans and M. Tyner in component 2, suggesting that this explains a weak component of H. J. Hancock or a component combining W. J. Evans and M. Tyner. In the sixth-order hierarchical model, the two components accounted for 10.269% of the total variance. In H. J. Hancock, the eigenvectors other than “The sorcerer” were lower than M. Tyner in component 1, suggesting that this explains a weak component of H. J. Hancock or M. Tyner. In W. J. Evans, the eigenvectors other than “I love you porgy” were lower than M. Tyner in component 2, suggesting that this explains a weak component of W. J. Evans or M. Tyner.

### Anova

#### Pitch sequence without rhythms

**Figure 3:**
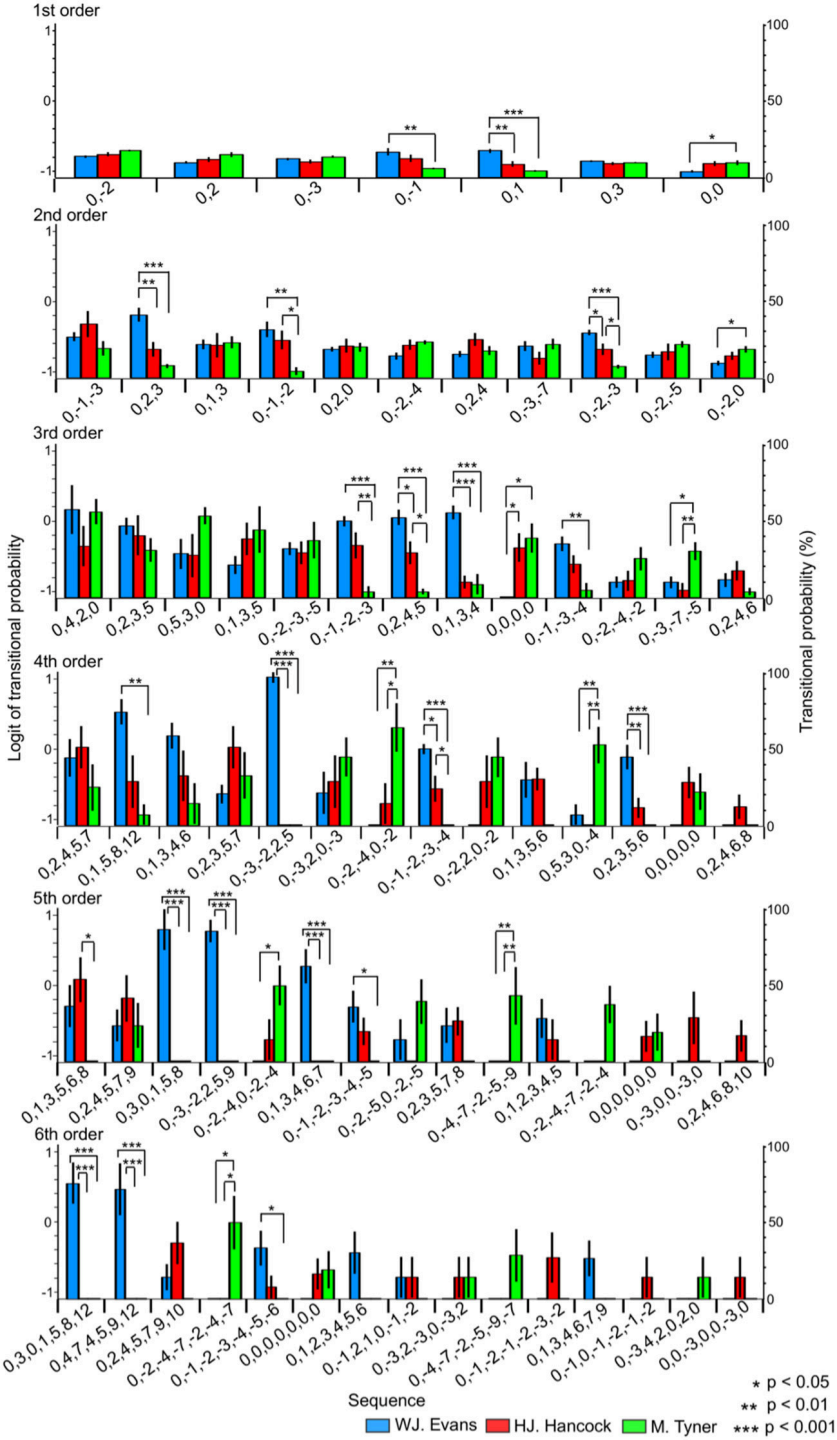
The difference in TPs among W.J. Evans (red), H.J. Hancock (blue), and M. Tyner (green) in pitch sequence without rhythms.

In the first-order hierarchical models, the main sequence effect were significant [*F*_(2.99, 53.84)_ = 7.51, *p* < 0.001, partial η^2^ = 0.29, Table 1A]. The main musician effect were significant [*F*_(2, 18)_ = 4.29, *p* = 0.030, partial η^2^ = 0.32]. The TPs in W. J. Evans were significantly higher than those in M. Tyner (*p* = 0.046). The musician-sequence interactions were significant [*F*_(12)_ = 6.54, *p* < 0.001, partial η^2^ = 0.42, Figure 3 and Tables 1B–D]. The TP of [0, −1] was significantly higher in W. J. Evans than M. Tyner (p = 0.008). The TP of [0, 0] was significantly lower in W. J. Evans than M. Tyner (*p* = 0.043). The TP of [0, 1] was significantly higher in W. J. Evans than H. J. Hancock (*p* = 0.003) and M. Tyner (*p* < 0.001). In the second-order hierarchical models, the main musician effect were significant [*F*_(2, 18)_ = 7.11, *p* = 0.005, partial η^2^ = 0.44]. The TPs in M. Tyner were significantly lower than those in W. J. Evans (*p* = 0.006) and H. J. Hancock (*p* = 0.041). The musician-sequence interactions were significant [*F*_(20)_ = 3.72, *p* < 0.001, partial η^2^ = 0.29, Figure 3 and Tables 1B–D]. The TP of [0, −1, −2] was significantly lower in M. Tyner than W. J. Evans (p = 0.006) and H. J. Hancock (*p* = 0.042). The TP of [0, −2, −3] was significantly higher in W. J. Evans than H. J. Hancock (*p* = 0.033) and M. Tyner (p < 0.001), and higher in H. J. Hancock than M. Tyner (*p* = 0.027). The TP of [0, −2, 0] was significantly higher in M. Tyner than W. J. Evans (*p* = 0.047). The TP of [0, 2, 3] was significantly higher in W. J. Evans than H. J. Hancock (*p* = 0.005) and M. Tyner (*p* < 0.001). In the third-order hierarchical models, the main sequence effect were significant [*F*_(5.13, 10.26)_ = 5.00, *p* < 0.001, partial η^2^ = 0.22, Table 1A]. The musician-sequence interactions were significant [*F*_(24)_ = 3.89, *p* < 0.001, partial η^2^ = 0.30, Figure 3 and Tables 1B–D]. The TP of [0, −1, −2, −3] was significantly lower in M. Tyner than W. J. Evans (*p* < 0.001) and H. J. Hancock (*p* = 0.008). The TP of [0, −1, −3, −4] was significantly lower in M. Tyner than W. J. Evans (*p* = 0.003). The TP of [0, −3, −7, −5] was significantly higher in M. Tyner than W. J. Evans (*p* = 0.040) and H. J. Hancock (*p* = 0.009). The TP of [0, 0, 0, 0] was significantly lower in W. J. Evans than H. J. Hancock (*p* = 0.037) and M. Tyner (*p* = 0.012). The TP of [0, 1, 3, 4] was significantly higher in W. J. Evans than H. J. Hancock (*p* < 0.001) and M. Tyner (*p* < 0.001). The TP of [0, 2, 4, 5] was significantly higher in W. J. Evans than H. J. Hancock (*p* = 0.034) and M. Tyner (*p* < 0.001), and higher in H. J. Hancock than M. Tyner (*p* = 0.021). In the forth-order hierarchical models, the main sequence effect were significant [*F*_(4.65, 9.30)_ = 2.40, *p* = 0.048, partial η^2^ = 0.12, Table 1A]. The musician-sequence interactions were significant [*F*_(26)_ = 5.92, *p* < 0.001, partial η^2^ = 0.40, Figure 3 and Tables 1B–D]. The TP of [0, −1, −2, −3, −4] was significantly higher in W. J. Evans than H. J. Hancock (*p* = 0.015) and M. Tyner (*p* < 0.001), and higher in H. J. Hancock than M. Tyner (*p* = 0.024). The TP of [0, −2, −4, 0, −2] was significantly higher in M. Tyner than W. J. Evans (*p* = 0.008) and H. J. Hancock (*p* = 0.042). The TP of [0, −3, −2, 2, 5] was significantly higher in W. J. Evans than H. J. Hancock (*p* < 0.001) and M. Tyner (*p* < 0.001). The TP of [0, 1, 5, 8, 12] was significantly higher in W. J. Evans than M. Tyner (*p* = 0.004). The TP of [0,2,3,5,6] was significantly higher in W. J. Evans than H. J. Hancock (*p* = 0.006) and M. Tyner (*p* < 0.001). The TP of [0, 5, 3, 0, −4] was significantly higher in M. Tyner than W. J. Evans (*p* = 0.004) and H. J. Hancock (*p* = 0.001). In W. J. Evans, the TPs of [0, −3, −2, 2, 5] was significantly higher than those of [0, −1, −2, −3, −4] (*p* < 0.001), [0, −2, −4,0, −2] (*p* < 0.001), [0, −2,2,0, −2] (*p* < 0.001), [0, −3,2,0, −3] (*p* = 0.021), [0, 0, 0, 0, 0] (*p* < 0.001), [0, 1, 3, 5, 6] (*p* < 0.001), [0, 2, 3, 5, 6] (*p* < 0.001), [0, 2, 3, 5, 7] (*p* = 0.002), [0, 2, 4, 6, 8] (*p* < 0.001), and [0,5,3,0, −4] (*p* < 0.001). The TPs of [0,0,0,0,0] was significantly lower than those of [0, −1, −2, −3, −4] (*p* = 0.002) and [0, 1, 5, 8, 12] (*p* = 0.045). The TPs of [0,2,4,6,8] was significantly lower than those of [0, −1, −2, −3, −4] (*p* < 0.001), [0, 1, 3, 4, 6] (*p* = 0.008), [0, 1, 5, 8, 12] (*p* = 0.003), and [0, 2, 3, 5, 6] (*p* = 0.008). In the fifth-order hierarchical models, the main musician effect were significant [*F*_(2, 18)_ = 4.13, *p* = 0.033, partial η^2^ = 0.32]. The TPs in M. Tyner were significantly lower than those in W. J. Evans (*p* = 0.006) and H. J. Hancock (*p* = 0.041). The musician-sequence interactions were significant [*F*_(28)_ = 7.07, *p* < 0.001, partial η^2^ = 0.44, Figure 3 and Tables 1B–D]. The TP of [0, −2, −4,−7, −2, −4] was significantly higher in M. Tyner than W. J. Evans (*p* = 0.008) and H. J. Hancock (*p* = 0.008). The TP of [0, −2, −4,0, −2, −4], [0, 1, 3, 4, 6, 7], and [0, 3, 0, 1, 5, 8] was significantly higher in M. Tyner than W. J. Evans (*p* = 0.022). The TP of [0, −3, −2, 2, 5, 9] was significantly higher in W. J. Evans than and H. J. Hancock and M. Tyner (all: *p* < 0.001). The TP of [0, 1, 3, 5, 6, 8] was significantly lower in H. J. Hancock than M. Tyner (*p* = 0.022). In the sixth-order hierarchical models, the musician-sequence interactions were significant [*F*_(28)_ = 5.09, *p* < 0.001, partial η^2^ = 0.36, Figure 3 and Tables 1B–D]. The TP of [0, −1, −2, −3, −4, −5, −6] was significantly lower in M. Tyner than W. J. Evans (*p* = 0.037). The TP of [0, −2, −4, −7, −2, −4, −7] was significantly higher in M. Tyner than W. J. Evans (*p* = 0.014) and H. J. Hancock (*p* = 0.014). The TP of [0, 3, 0, 1, 5, 8, 12] and [0, 4, 7, 4, 5, 9, 12] was significantly higher in W. J. Evans than H. J. Hancock and M. Tyner (*p* < 0.001).

**Table 1:** The difference in TPs among pitch sequences without rhythms in each musician.

**Order**	**Sequence A**	**Sequence B**	**A-B**	**SE**	***p*-value**
**A. GENERAL**					
1st	0, −2	0, −3	0.076	0.02	0.023
		0, 0	0.173	0.02	<0.001
		0, 1	0.116	0.03	0.028
		0, 3	0.122	0.014	<0.001
	0, −3	0,0	0.097	0.02	0.002
		0,3	0.045	0.01	0.005
3rd	0, −3, −7, −5	0, 4, 2, 0	−0.742	0.159	0.015
	0, 2, 4, 6	0, −1, −2, −3	−0.398	0.079	0.007
		0, 1, 3, 5	−0.519	0.152	0.24
		0, 2, 3, 5	−0.621	0.11	0.002
		0, 2, 4, 5	−0.383	0.068	0.002
		0, 4, 2, 0	−0.831	0.176	0.014
4th	0, 2, 4, 6, 8	0, −1, −2, −3, −4	−0.452	0.077	0.001
		0, −3, −2,2,5	−0.616	0.068	<0.001
		0, 1, 3, 4, 6	−0.68	0.147	0.019
		0, 1, 5, 8, 12	−0.714	0.169	0.046
		0, 2, 4, 5, 7	−0.786	0.184	0.041
**B. W. J. EVANS**					
1st	0, 0	0, −1	−0.279	0.069	0.016
		0, −2	−0.213	0.035	<0.001
		0, −3	−0.177	0.034	0.001
		0, 1	−0.294	0.053	0.001
		0, 3	−0.149	0.034	0.007
2nd	0, −2, −3	0, −2, 0	0.435	0.09	0.007
	0,2,3	0, −2, −4	0.585	0.124	0.009
		0, −2, 0	0.688	0.115	0.001
		0,2,4	0.557	0.12	0.012
3rd	0, −1, −2, −3	0, −3, −7, −5	0.869	0.2	0.03
		0, 0, 0, 0	1.09	0.208	0.004
		0, 2, 4, 6	0.838	0.138	0.001
	0, 1, 3, 4	0, −1, −3, −4	0.442	0.099	0.023
		0, −2, −4, −2	0.991	0.152	<0.001
		0, −3, −7, −5	0.992	0.149	<0.001
		0, 0, 0, 0	1.214	0.234	0.005
		0, 2, 4, 6	0.961	0.147	<0.001
	0, 2, 4, 5	0, −2, −4, −2	0.924	0.209	0.026
		0, −3, −7, −5	0.925	0.196	0.013
		0, 0, 0, 0	1.147	0.226	0.006
		0, 2, 4, 6	0.894	0.118	<0.001
4th	0, −3, −2,2,5	0, −1, −2, −3, −4	1.019	0.122	<0.001
		0, −2, −4, 0, −2	2.113	0.287	<0.001
		0, −2, 2, 0, −2	2.113	0.297	<0.001
		0, −3, 2, 0, −3	1.642	0.358	0.021
		0, 0, 0, 0, 0	2.113	0.219	<0.001
		0, 1, 3, 5, 6	1.459	0.212	<0.001
		0, 2, 3, 5, 6	1.134	0.164	<0.001
		0, 2, 3, 5, 7	1.663	0.296	0.002
		0, 2, 4, 6, 8	2.113	0.119	<0.001
		0, 5, 3, 0, −4	1.956	0.188	<0.001
	0, 0, 0, 0, 0	0, −1, −2, −3, −4	−1.094	0.19	0.002
		0, 1, 5, 8, 12	−1.623	0.383	0.045
	0, 2, 4, 6, 8	0, −1, −2, −3, −4	−1.094	0.134	<0.001
		0, 1, 3, 4, 6	−1.28	0.255	0.008
		0, 1, 5, 8, 12	−1.623	0.293	0.003
		0, 2, 3, 5, 6	−0.979	0.194	0.008
5th	0, −3, −2, 2, 5, 9	0, −1, −2, −3, −4, −5	1.077	0.183	0.002
		0, −2, −4, −7, −2, −4	1.863	0.194	<0.001
		0, −2, −4, 0, −2, −4	1.863	0.272	<0.001
		0, −2, −5, 0, −2, −5	1.549	0.264	0.002
		0, −3, 0, 0, −3, 0	1.863	0.254	<0.001
		0, −4, −7, −2, −5, −9	1.863	0.275	<0.001
		0, 0, 0, 0, 0, 0	1.863	0.237	<0.001
		0, 1, 2, 3, 4, 5	1.246	0.222	0.003
		0, 2, 3, 5, 7, 8	1.344	0.231	0.002
		0, 2, 4, 6, 8, 10	1.863	0.172	<0.001
	0, 1, 3, 4, 6, 7	0, −2, −4, −7,−2,−4	1.361	0.228	0.001
		0, −2, −4, 0, −2, −4	1.361	0.297	0.024
		0, −3, 0, 0, −3, 0	1.361	0.28	0.013
		0, −4, −7, −2, −5, −9	1.361	0.299	0.026
		0, 0, 0, 0, 0, 0	1.361	0.265	0.007
		0, 2, 4, 6, 8, 10	1.361	0.209	<0.001
	0, 3, 0, 1, 5, 8	0, −2, −4, −7, −2, −4	1.883	0.247	<0.001
		0, −2, −4, 0, −2, −4	1.883	0.312	0.001
		0, −2, −5, 0, −2, −5	1.569	0.306	0.007
		0, −3, 0, 0, −3,0	1.883	0.296	0.001
		0, −4, −7, −2, −5, −9	1.883	0.314	0.001
		0, 0, 0, 0, 0, 0	1.883	0.281	<0.001
		0, 2, 3, 5, 7, 8	1.364	0.236	0.002
		0, 2, 4, 6, 8, 10	1.883	0.23	<0.001
6th	0, 3, 0, 1, 5, 8, 12	0, −1, −2, −1, −2, −3, −2	1.658	0.289	0.002
		0, −1, −2, 1, 0, −1, −2	1.344	0.284	0.017
		0, −1, 0, −1, −2, −1, −2	1.658	0.258	<0.001
		0, −2, −4, −7, −2, −4, −7	1.658	0.302	0.003
		0, −3, 2, −3, 0, −3, 2	1.658	0.315	0.006
		0, −3, 4, 2, 0, 2, 0	1.658	0.258	<0.001
		0, −4, −7, −2, −5, −9, −7	1.658	0.297	0.003
		0, 0, −3, 0, 0, −3, 0	1.658	0.258	<0.001
		0, 0, 0, 0, 0, 0, 0	1.658	0.281	0.001
		0, 1, 3, 4, 6, 7, 9	1.07	0.187	0.002
		0, 2, 4, 5, 7, 9, 10	1.344	0.269	0.01
	0, 4, 7, 4, 5, 9, 12	0, −1, −2, −1, −2, −3, −2	1.569	0.323	0.013
		0, −1, 0, −1, −2, −1, −2	1.569	0.296	0.005
		0, −2, −4, −7, −2, −4, −7	1.569	0.335	0.019
		0, −3, 2, −3, 0, −3, 2	1.569	0.347	0.028
		0, −3, 4, 2, 0, 2, 0	1.569	0.296	0.005
		0, −4, −7, −2, −5, −9, −7	1.569	0.331	0.017
		0, 0,−3, 0, 0, −3,0	1.569	0.296	0.005
		0, 0, 0, 0, 0, 0, 0	1.569	0.316	0.011
		0, 1, 3, 4, 6, 7, 9	0.982	0.199	0.011
		0, 2, 4, 5, 7, 9, 10	1.256	0.286	0.038
**C. H. J. HANCOCK**					
1st	0, −2	0, 0	0.132	0.035	0.029
		0, 3	0.13	0.024	0.001
4th	0, −1, −2, −3, −4	0, −3, −2, 2, 5	0.529	0.122	0.036
**D. M. TYNER**					
1st	0, −2	0, −1	0.255	0.06	0.01
		0, 0	0.175	0.035	0.002
		0, 1	0.29	0.053	0.001
		0, 3	0.171	0.024	<0.001
	0, −3	0,3	0.084	0.017	0.002
	0,1	0, −3	−0.202	0.05	0.016
		0,2	−0.231	0.049	0.003
3rd	0, −1, −2, −3	0, 1, 3, 5	−0.889	0.211	0.04
	0, −1, −3, −4	0, −2, −3, −5	−0.713	0.165	0.033
4th	0,−2,−4,0,−2	0, −1, −2, −3, −4	1.402	0.266	0.005
		0, −3, −2, 2, 5	1.402	0.287	0.011
		0, 1, 3, 5, 6	1.402	0.312	0.026
		0, 2, 3, 5, 6	1.402	0.33	0.044
		0, 2, 4, 6, 8	1.402	0.237	0.001
	0, 5, 3, 0, −4	0, −1, −2, −3, −4	1.148	0.23	0.009
		0, −3, −2, 2, 5	1.148	0.188	0.001
		0, 1, 3, 5, 6	1.148	0.255	0.025
		0, 2, 3, 5, 6	1.148	0.242	0.015
		0, 2, 4, 6, 8	1.148	0.214	0.004
5th	0, −2, −4, 0, −2, −4	0, 2, 4, 6, 8, 10	1.086	0.211	0.007

#### Rhythm sequence without pitches

**Figure 4:**
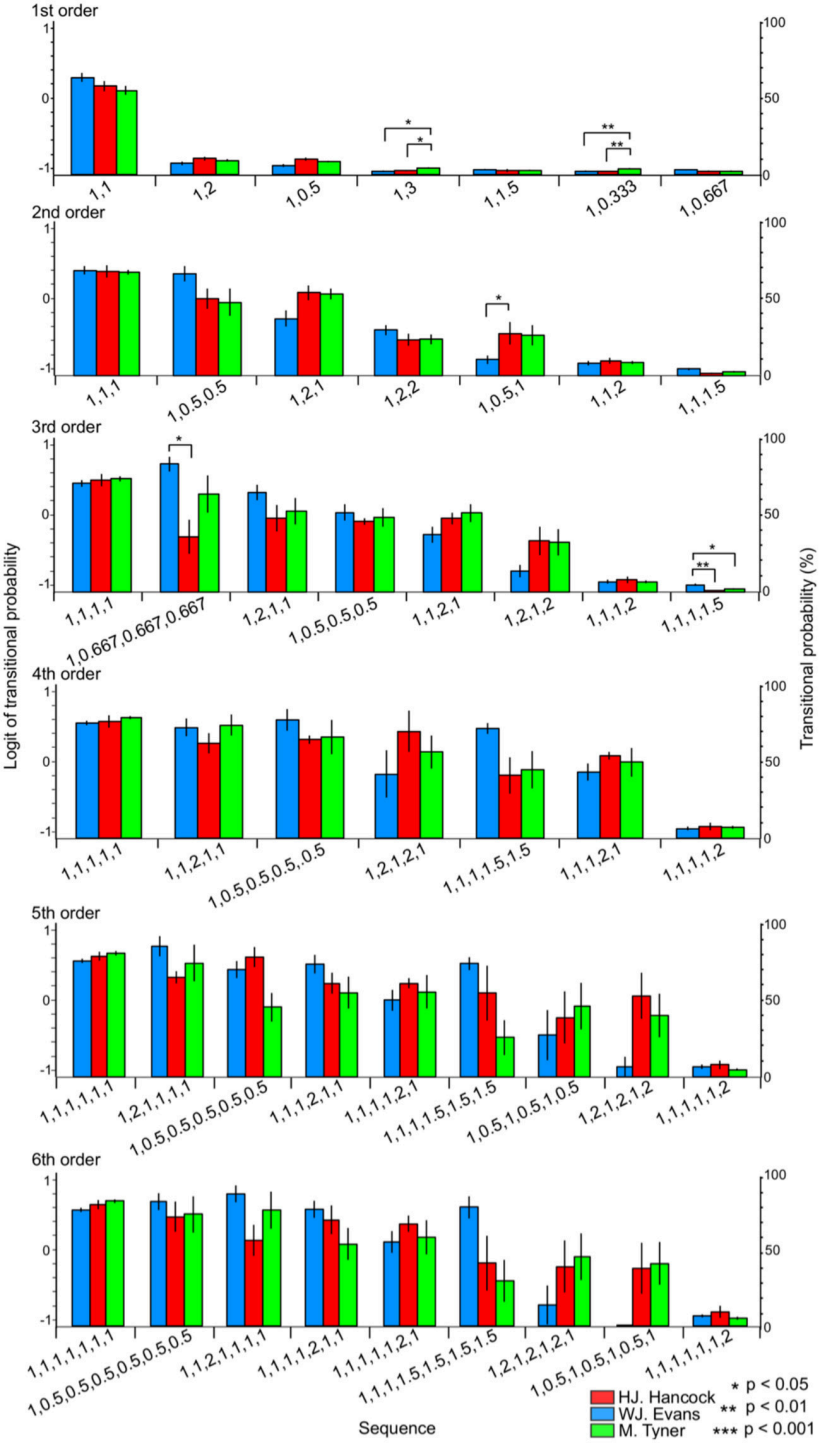
The difference in TPs among W.J. Evans (red), H.J. Hancock (blue), and M. Tyner (green) in rhythm sequences without pitches.

In the first-order hierarchical models, the main sequence effect were significant [*F*_(1.24, 22.36)_ = 553.50, *p* < 0.001, partial η^2^ = 0.97, Table 2A]. The musician-sequence interactions were significant [*F*_(12)_ = 2.03, *p* = 0.028, partial η^2^ = 0.18, Figure 4, Tables 2B–D]. The TP of [1, 3] was significantly higher in M. Tyner than W. J. Evans (*p* = 0.015) and H. J. Hancock (*p* = 0.023). The TP of [1, 0.333] was significantly higher in M. Tyner than W. J. Evans (*p* = 0.006) and H. J. Hancock (*p* = 0.002). In the second-order hierarchical models, the main sequence effect were significant [*F*_(2.09, 37.68)_ = 74.54, *p* < 0.001, partial η^2^ = 0.81, Table 2A]. The musician-sequence interactions were significant [*F*_(12)_ = 2.07, *p* = 0.025, partial η^2^ = 0.19, Figure 4, Tables 2B–D]. The TP of [1, 0.333] was significantly higher in H. J. Hancock than W. J. Evans (*p* = 0.015). In W. J. Evans, the TPs of [1, 1, 1] was significantly higher than those of [1, 0.5, 1], [1, 1, 1.5], [1, 1, 2], [1, 2, 1], and [1, 2, 2] (all: *p* < 0.001). The TPs of [1,0.5,0.5] was significantly higher than those of [1, 0.5, 1] (*p* = 0.013), [1,1,1.5] (*p* < 0.001), [1, 1, 2] (*p* < 0.001), and [1, 2, 2] (*p* = 0.003). The TPs of [1, 2, 1] was significantly higher than [1, 0.5, 1] (*p* = 0.034), [1, 1, 1.5] (*p* < 0.001), and [1, 1, 2] (*p* = 0.001). The TPs of [1, 2, 1] was significantly higher than [1, 1, 1.5] (*p* < 0.001) and [1, 1, 2] (*p* < 0.001). In H. J. Hancock, the TPs of [1, 1, 1] was significantly higher than those of [1, 0.5, 1], [1, 1, 1.5], [1, 1, 2], [1, 2, 1], and [1, 2, 2] (all: *p* < 0.001). The TPs of [1, 0.5, 0.5] was significantly higher than those of [1, 1, 1.5] (*p* < 0.001) and [1, 1, 2] (*p* = 0.001). The TPs of [1, 2, 1] was significantly higher than [1, 2, 2] (*p* = 0.027), [1, 0.5, 1] (*p* = 0.038), [1, 1, 1.5] (*p* < 0.001), and [1, 1, 2] (*p* < 0.001). The TPs of [1, 2, 2] was significantly higher than [1, 1, 1.5] (*p* < 0.001) and [1, 1, 2] (*p* = 0.037). The TPs of [1, 1, 1.5] was significantly lower than those of [1, 1, 2] (*p* = 0.006) and [1, 0.5, 1] (*p* = 0.015). In the third-order hierarchical models, the main sequence effect were significant [*F*_(2.80, 50.41)_ = 45.17, *p* < 0.001, partial η^2^ = 0.72, Table 2A]. The musician-sequence interactions were significant [*F*_(14)_ = 2.58, *p* = 0.03, partial η^2^ = 0.22, Figure 4, Tables 2B–D]. The TP of [1,0.667, 0.667, 0.667] was significantly higher in W. J. Evans than H. J. Hancock (*p* = 0.016). The TP of [1, 1, 1, 1.5] was significantly higher in W. J. Evans than H. J. Hancock (*p* = 0.002) and M. Tyner (*p* = 0.043). In the forth-order hierarchical models, the main sequence effect were significant [*F*_(2.62, 47.21)_ = 22.03, *p* < 0.001, partial η^2^ = 0.55, Table 2A]. In the fifth-order hierarchical models, the main sequence effect were significant [*F*_(3.02, 54.32)_ = 16.21, *p* < 0.001, partial η^2^ = 0.47, Table 2A]. The musician-sequence interactions were significant [*F*_(16)_ = 2.11, *p* = 0.011, partial η^2^ = 0.19, Figure 4, Tables 2B–D]. In the sixth-order hierarchical models, the main sequence effect were significant [*F*_(3.28, 59.06)_ = 17.89, *p* < 0.001, partial η^2^ = 0.50, Table 2A]. The musician-sequence interactions were significant [*F*_(16)_ = 2.22, *p* = 0.007, partial η^2^ = 0.20, Figure 4 and Tables 2B–D].

**Table 2:** The difference in TPs among rhythm sequences without pitches in each musician.

**Order**	**Sequence A**	**Sequence B**	**A-B**	**SE**	***p*-value**
**A. GENERAL**					
1st	1, 1	1, 2	1.088	0.054	<0.001
		1, 0.5	1.105	0.051	<0.001
		1, 1.5	1.221	0.047	<0.001
		1, 0.667	1.229	0.043	<0.001
		1, 3	1.218	0.043	<0.001
		1, 0.333	1.226	0.043	<0.001
	1, 2	1,1.5	0.133	0.014	<0.001
		1, 0.667	0.141	0.015	<0.001
		1, 3	0.13	0.015	<0.001
		1, 0.333	0.138	0.015	<0.001
	1, 0.5	1,1.5	0.116	0.013	<0.001
		1, 0.667	0.124	0.014	<0.001
		1, 3	0.113	0.014	<0.001
		1, 0.333	0.121	0.014	<0.001
2nd	1, 0.5, 1	1, 0.5, 0.5	−0.764	0.179	0.01
		1, 2, 1	−0.612	0.096	<0.001
	1, 1, 1	1,0.5,1	1.065	0.093	<0.001
		1, 1, 1.5	1.495	0.044	<0.001
		1, 1, 2	1.353	0.061	<0.001
		1, 2, 1	0.453	0.065	<0.001
		1, 2, 2	0.969	0.071	<0.001
	1, 1, 1.5	1, 0.5, 0.5	−1.194	0.102	<0.001
		1, 0.5, 1	−0.43	0.085	0.002
		1, 1, 2	−0.142	0.025	<0.001
		1, 2, 1	−1.042	0.067	<0.001
		1, 2, 2	−0.526	0.051	<0.001
	1, 1, 2	1, 0.5, 0.5	−1.052	0.102	<0.001
		1, 2, 1	−0.9	0.074	<0.001
		1, 2, 2	−0.384	0.049	<0.001
	1, 2, 1	1,2,2	0.516	0.107	0.003
	1, 2, 2	1, 0.5, 0.5	−0.667	0.1	<0.001
3rd	1, 0.5, 0.5, 0.5	1, 1, 1, 1.5	1.062	0.072	<0.001
		1, 1, 1,2	0.955	0.073	<0.001
	1, 0.667, 0.667, 0.667	1, 1, 1, 1.5	1.343	0.139	<0.001
		1, 1, 1, 2	1.236	0.153	<0.001
		1, 2, 1, 2	0.794	0.168	0.005
	1,1, 1, 1	1, 0.5, 0.5,0.5	0.542	0.07	<0.001
		1, 1, 1, 1.5	1.605	0.043	<0.001
		1, 1, 1, 2	1.497	0.058	<0.001
		1, 1, 2, 1	0.616	0.073	<0.001
		1, 2, 1, 2	1.055	0.115	<0.001
	1, 1, 1, 2	1, 1, 1, 1.5	0.107	0.025	0.012
	1, 1, 2, 1	1, 1, 1, 1.5	0.989	0.076	<0.001
		1, 1, 1, 2	0.882	0.075	<0.001
	1, 2, 1, 1	1, 1, 1, 1.5	1.209	0.112	<0.001
		1, 1, 1, 2	1.101	0.114	<0.001
	1, 2, 1, 2	1, 1, 1, 1.5	0.55	0.11	0.003
		1, 1, 1, 2	0.442	0.117	0.037
4th	1, 0.5, 0.5, 0.5, 0.5	1, 1, 1, 2, 1	0.464	0.074	<0.001
	1, 1, 1, 1, 1	1, 1, 1, 1.5, 1.5	0.557	0.156	0.046
		1, 1, 1, 2, 1	0.635	0.095	<0.001
	1, 1, 1, 1, 2	1, 0.5, 0.5,0.5,0.5	−1.423	0.11	<0.001
		1, 1, 1, 1, 1	−1.594	0.059	<0.001
		1, 1, 1, 1.5, 1.5	−1.037	0.136	<0.001
		1, 1, 1, 2, 1	−0.959	0.093	<0.001
		1, 1, 2, 1, 1	−1.425	0.108	<0.001
		1, 2, 1, 2, 1	−1.119	0.201	0.001
5th	1, 1, 1, 1, 1,1	1,0.5,1,0.5,1,0.5	0.939	0.226	0.022
		1, 1, 1, 1, 1, 2	1.648	0.055	<0.001
		1, 1, 1, 1, 2, 1	0.524	0.108	0.004
		1, 2, 1, 2, 1, 2	1.035	0.171	<0.001
	1, 1, 1, 1, 1, 2	1, 0.5, 0.5,0.5,0.5,0.5	−1.33	0.116	<0.001
		1, 1, 1, 1, 1,1	−1.648	0.055	<0.001
		1, 1, 1, 1, 2, 1	−1.124	0.111	<0.001
		1, 1, 1, 1.5, 1.5, 1.5	−1.03	0.195	0.002
		1, 1, 1, 2, 1,1	−1.299	0.126	<0.001
		1, 2, 1, 1, 1, 1	−1.567	0.12	<0.001
	1, 2, 1, 1, 1, 1	1, 1, 1, 1, 2, 1	0.443	0.112	0.033
		1, 2, 1, 2, 1, 2	0.953	0.238	0.03
6th	1, 0.5, 1, 0.5, 1, 0.5, 1	1, 0.5, 0.5, 0.5, 0.5, 0.5, 0.5	−1.122	0.208	0.001
		1, 1, 1, 1, 1,1,1	−1.206	0.17	<0.001
		1, 1, 2, 1, 1, 1, 1	−1.064	0.208	0.003
	1, 1, 1, 1, 1, 1, 2	1, 0.5, 0.5, 0.5, 0.5, 0.5, 0.5	−1.56	0.154	<0.001
		1, 1, 1, 1, 1,1,1	−1.644	0.064	<0.001
**B. W. J. EVANS**					
1st	1, 1	1,2	1.23	0.094	<0.001
		1, 0.5	1.255	0.089	<0.001
		1, 1.5	1.313	0.081	<0.001
		1, 0.667	1.324	0.075	<0.001
		1, 3	1.337	0.074	<0.001
		1, 0.333	1.34	0.075	<0.001
	1, 2	1,0.667	0.093	0.026	0.047
		1, 3	0.107	0.027	0.017
		1, 0.333	0.11	0.025	0.009
2nd	1, 0.5, 0.5	1,0.5,1	1.287	0.31	0.013
		1, 1, 1.5	1.426	0.178	<0.001
		1, 1, 2	1.337	0.177	<0.001
		1, 2, 2	0.843	0.173	<0.001
	1,1, 1	1,0.5,1	1.334	0.162	<0.001
		1, 1, 1.5	1.473	0.076	<0.001
		1, 1, 2	1.384	0.106	<0.001
		1, 2, 1	0.72	0.113	<0.001
		1, 2, 2	0.891	0.123	<0.001
	1, 2, 1	1,0.5,1	0.614	0.166	0.034
		1, 1, 1.5	0.753	0.117	<0.001
		1, 1, 2	0.665	0.129	<0.001
	1, 2, 2	1, 1, 1.5	0.582	0.088	<0.001
		1, 1, 2	0.494	0.085	<0.001
3rd	1, 0.5, 0.5, 0.5	1, 1, 1, 1.5	1.084	0.125	<0.001
		1, 1, 1, 2	1.034	0.126	<0.001
	1,0.667,0.667,0.667	1, 1, 1, 1.5	1.809	0.241	<0.001
		1, 1, 1, 2	1.76	0.265	<0.001
		1, 1, 2, 1	1.056	0.253	0.016
		1, 2, 1, 2	1.602	0.291	0.001
	1, 1, 1, 1	1, 1, 1, 1.5	1.517	0.074	<0.001
		1, 1, 1, 2	1.468	0.101	<0.001
		1, 1, 2, 1	0.764	0.126	<0.001
		1, 2, 1, 2	1.31	0.199	<0.001
	1, 1, 2, 1	1, 1, 1, 1.5	0.753	0.131	0.001
		1, 1, 1, 2	0.704	0.129	0.001
	1, 2, 1, 1	1, 1, 1, 1.5	1.38	0.194	<0.001
		1, 1, 1, 2	1.331	0.198	<0.001
4th	1, 1, 1, 1, 2	1, 0.5, 0.5,0.5,0.5	−1.623	0.191	<0.001
		1, 1, 1, 1, 1	−1.579	0.102	<0.001
		1, 1, 1, 1.5, 1.5	−1.488	0.235	<0.001
		1, 1, 1, 2, 1	−0.843	0.161	0.001
		1, 1, 2, 1, 1	−1.507	0.187	<0.001
	1, 1, 1, 2, 1	1, 0.5, 0.5,0.5,0.5	−0.78	0.129	<0.001
		1, 1, 1, 1, 1	−0.736	0.164	0.006
		1, 1, 1, 1, 2	0.843	0.161	0.001
5th	1, 1, 1, 1, 1,2	1, 0.5, 0.5,0.5,0.5,0.5	−1.451	0.201	<0.001
		1, 1, 1, 1, 1, 1	−1.584	0.095	<0.001
		1, 1, 1, 1, 2, 1	−0.993	0.192	0.002
		1, 1, 1, 1.5, 1.5, 1.5	−1.54	0.338	0.009
		1, 1, 1, 2, 1,1	−1.532	0.217	<0.001
		1, 2, 1, 1, 1, 1	−1.801	0.208	<0.001
	1, 1, 1, 1, 2, 1	1, 2, 1, 1, 1, 1	−0.809	0.194	0.02
	1, 2, 1, 2, 1, 2	1, 0.5, 0.5,0.5,0.5,0.5	−1.449	0.361	0.03
		1, 1, 1, 1, 1, 1	−1.582	0.296	0.002
		1, 1, 1, 1.5, 1.5, 1.5	−1.538	0.317	0.005
		1, 1, 1, 2, 1, 1	−1.53	0.403	0.048
		1, 2, 1, 1, 1, 1	−1.799	0.412	0.013
6th	1, 0.5, 1, 0.5, 1, 0.5, 1	1, 0.5, 0.5, 0.5, 0.5, 0.5, 0.5	−1.862	0.36	0.002
		1, 1, 1, 1, 1, 1, 1	−1.739	0.294	<0.001
		1, 1, 1, 1, 2, 1,1	−1.743	0.442	0.034
		1, 1, 1, 1.5, 1.5, 1.5,1.5	−1.777	0.434	0.024
		1,1, 2, 1, 1, 1, 1	−1.979	0.36	0.001
	1, 1, 1, 1, 1, 1, 2	1, 0.5, 0.5, 0.5, 0.5, 0.5, 0.5	−1.708	0.266	<0.001
		1, 1, 1, 1, 1, 1, 1	−1.585	0.11	<0.001
		1, 1, 1, 1, 1, 2, 1	−1.103	0.213	0.002
		1, 1, 1, 1, 2, 1, 1	−1.589	0.24	<0.001
		1, 1, 1, 1.5, 1.5, 1.5,1.5	−1.623	0.37	0.013
		1, 1, 2, 1, 1, 1, 1	−1.826	0.259	<0.001
	1, 2, 1, 2, 1, 2, 1	1, 0.5, 0.5, 0.5, 0.5, 0.5, 0.5	−1.548	0.396	0.037
		1, 1, 1, 1, 1, 1, 1	−1.425	0.364	0.036
		1, 1, 2, 1, 1, 1, 1	−1.666	0.431	0.041
**C. H. J. HANCOCK**					
1st	1, 1	1, 2	1.035	0.094	<0.001
		1, 0.5	1.045	0.089	<0.001
		1, 1.5	1.205	0.081	<0.001
		1, 0.667	1.214	0.075	<0.001
		1, 3	1.209	0.074	<0.001
		1, 0.333	1.221	0.075	<0.001
	1, 2	1,1.5	0.17	0.024	<0.001
		1, 0.667	0.179	0.026	<0.001
		1, 3	0.174	0.027	<0.001
		1, 0.333	0.186	0.025	<0.001
	1, 0.5	1,1.5	0.16	0.023	<0.001
		1, 0.667	0.169	0.024	<0.001
		1, 3	0.164	0.025	<0.001
		1, 0.333	0.176	0.024	<0.001
2nd	1, 0.5, 0.5	1, 1, 1.5	1.115	0.178	<0.001
		1, 1, 2	0.92	0.177	0.001
	1,0.5,1	1, 1, 1.5	0.602	0.148	0.015
	1,1,1	1,0.5,1	0.924	0.162	<0.001
		1, 1, 1.5	1.527	0.076	<0.001
		1, 1, 2	1.331	0.106	<0.001
		1, 2, 2	1.02	0.123	<0.001
	1, 1, 2	1, 1, 1.5	0.195	0.044	0.006
	1, 2, 1	1,0.5,1	0.606	0.166	0.038
		1, 1, 1.5	1.208	0.117	<0.001
		1, 1, 2	1.013	0.129	<0.001
		1, 2, 2	0.701	0.185	0.027
	1, 2, 2	1, 1, 1.5	0.507	0.088	<0.001
		1, 1, 2	0.312	0.085	0.037
3rd	1, 0.5, 0.5,0.5	1, 1, 1, 1.5	1.037	0.125	<0.001
		1, 1, 1, 2	0.871	0.126	<0.001
	1, 1, 1, 1	1, 0.5, 0.5,0.5	0.615	0.122	0.002
		1, 1, 1, 1.5	1.651	0.074	<0.001
		1, 1, 1, 2	1.485	0.101	<0.001
		1, 1, 2, 1	0.571	0.126	0.007
		1, 2, 1, 2	0.906	0.199	0.007
	1, 1, 2, 1	1, 1, 1, 1.5	1.081	0.131	<0.001
		1, 1, 1, 2	0.915	0.129	<0.001
	1, 2, 1, 1	1, 1, 1, 1.5	1.085	0.194	0.001
		1, 1, 1, 2	0.919	0.198	0.006
	1, 2, 1, 2	1, 1, 1, 1	−0.906	0.199	0.007
		1, 1, 1, 1.5	0.745	0.19	0.028
4th	1, 1, 1, 1, 2	1, 0.5, 0.5,0.5,0.5	−1.294	0.191	<0.001
		1, 1, 1, 1, 1	−1.565	0.102	<0.001
		1, 1, 1, 2, 1	−1.059	0.161	<0.001
		1, 1, 2, 1, 1	−1.239	0.187	<0.001
		1, 2, 1, 2, 1	−1.418	0.349	0.015
5th	1, 1, 1, 1, 1, 2	1, 0.5, 0.5,0.5,0.5,0.5	−1.608	0.201	<0.001
		1, 1, 1, 1, 1,1	−1.622	0.095	<0.001
		1, 1, 1, 1, 2, 1	−1.218	0.192	<0.001
		1, 1, 1, 2, 1, 1	−1.217	0.217	0.001
		1, 2, 1, 1, 1, 1	−1.309	0.208	<0.001
6th	1, 1, 1, 1, 1, 1, 2	1, 0.5, 0.5, 0.5, 0.5, 0.5, 0.5	−1.418	0.266	0.002
		1, 1, 1, 1, 1,1,1	−1.597	0.11	<0.001
**D. M. TYNER**					
		1, 1, 1, 1, 1, 2, 1	−1.31	0.213	<0.001
		1, 1, 1, 1, 2, 1,1	−1.37	0.24	0.001
		1, 1, 2, 1, 1, 1, 1	−1.066	0.259	0.023
1st	1, 1	1,2	0.999	0.094	<0.001
		1, 0.5	1.014	0.089	<0.001
		1, 1.5	1.144	0.081	<0.001
		1, 0.667	1.149	0.075	<0.001
		1, 3	1.106	0.074	<0.001
		1, 0.333	1.117	0.075	<0.001
	1, 2	1,1.5	0.145	0.024	<0.001
		1, 0.667	0.15	0.026	<0.001
		1, 3	0.108	0.027	0.016
		1, 0.333	0.118	0.025	0.004
2nd	1, 0.5, 0.5	1, 1, 1.5	1.041	0.178	<0.001
		1, 1, 2	0.898	0.177	0.002
	1, 0.5,1	1, 1, 1.5	0.55	0.148	0.033
	1, 1, 1	1, 0.5, 1	0.936	0.162	<0.001
		1, 1, 1.5	1.486	0.076	<0.001
		1, 1, 2	1.342	0.106	<0.001
		1, 2, 2	0.995	0.123	<0.001
	1, 2, 1	1,0.5,1	0.615	0.166	0.034
		1, 1, 1.5	1.165	0.117	<0.001
		1, 1, 2	1.021	0.129	<0.001
		1, 2, 2	0.674	0.185	0.038
	1, 2, 2	1, 1, 1.5	0.491	0.088	0.001
		1, 1, 2	0.347	0.085	0.015
3rd	1, 0.5, 0.5,0.5	1, 1, 1, 1	−0.579	0.122	0.004
		1, 1, 1, 1.5	1.067	0.125	<0.001
		1, 1, 1, 2	0.96	0.126	<0.001
	1,0.667,0.667,0.667	1, 1, 1, 1.5	1.417	0.241	<0.001
		1, 1, 1, 2	1.31	0.265	0.003
	1, 1, 1, 1	1, 0.5, 0.5,0.5	0.579	0.122	0.004
		1, 1, 1, 1.5	1.646	0.074	<0.001
		1, 1, 1, 2	1.539	0.101	<0.001
		1, 1, 2, 1	0.513	0.126	0.02
		1, 2, 1, 2	0.95	0.199	0.004
	1, 1, 2, 1	1, 1, 1, 1.5	1.134	0.131	<0.001
		1, 1, 1, 2	1.027	0.129	<0.001
	1, 2, 1, 1	1, 1, 1, 1.5	1.161	0.194	<0.001
		1, 1, 1, 2	1.054	0.198	0.001
4th	1, 1, 1, 1, 2	1, 0.5, 0.5,0.5,0.5	−1.352	0.191	<0.001
		1, 1, 1, 1, 1	−1.638	0.102	<0.001
		1, 1, 1, 1.5, 1.5	−0.86	0.235	0.038
		1, 1, 1, 2, 1	−0.976	0.161	<0.001
		1, 1, 2, 1, 1	−1.529	0.187	<0.001
	1, 1, 1, 2, 1	1, 1, 1, 1, 1	−0.662	0.164	0.017
5th	1, 1, 1, 1, 1,1	1, 0.5, 0.5,0.5,0.5,0.5	0.805	0.164	0.004
		1, 1, 1, 1.5, 1.5, 1.5	1.258	0.287	0.013
	1, 1, 1, 1, 1,2	1, 0.5, 0.5,0.5,0.5,0.5	−0.932	0.201	0.007
		1, 1, 1, 1, 1,1	−1.737	0.095	<0.001
		1, 1, 1, 1, 2, 1	−1.161	0.192	<0.001
		1, 1, 1, 2, 1,1	−1.147	0.217	0.002
		1, 2, 1, 1, 1, 1	−1.59	0.208	<0.001
6th	1, 1, 1, 1, 1, 1, 2	1, 0.5, 0.5, 0.5, 0.5, 0.5, 0.5	−1.555	0.266	0.001
		1, 1, 1, 1, 1, 1, 1	−1.751	0.11	<0.001
		1, 1, 1, 1, 1, 2, 1	−1.212	0.213	0.001
		1, 1, 1, 1, 2, 1,1	−1.11	0.24	0.008
		1, 1, 2, 1, 1, 1, 1	−1.614	0.259	<0.001

#### Pitch sequence with rhythms

**Figure 5:**
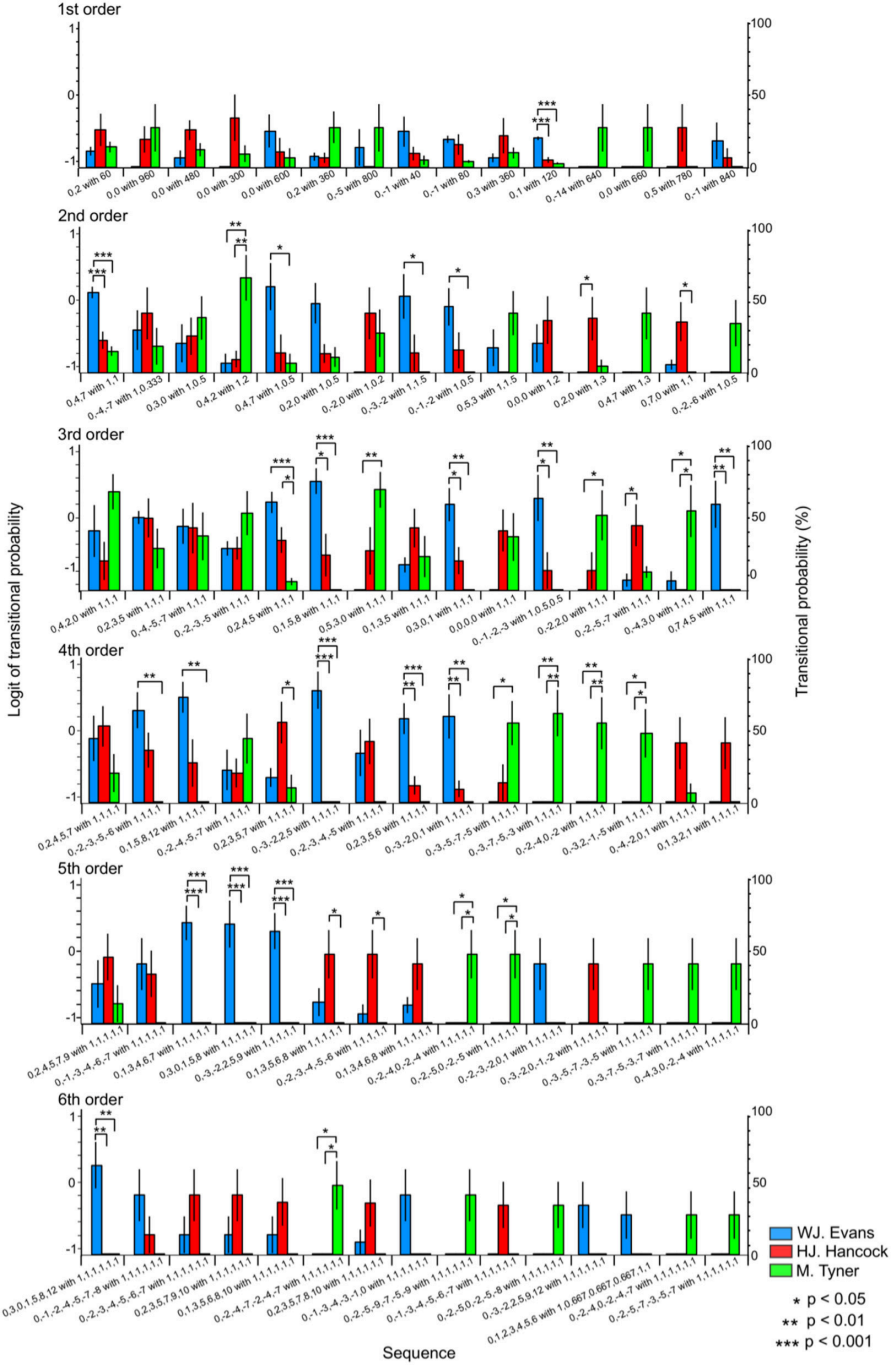
The difference in TPs among W.J. Evans (red), H.J. Hancock (blue), and M. Tyner (green) in pitch sequence with rhythms.

The relative pattern of Pitch sequence (p) with rhythms (r) were represented as [p] with [r]. In the first-order hierarchical models, the musician-sequence interactions were significant [*F*_(28)_ = 1.89, *p* = 0.006, partial η^2^ = 0.17, Figure 5]. The TP of [0, 1] with [0.5] was significantly higher in W. J. Evans than H. J. Hancock and M. Tyner (*p* < 0.001). In the second-order hierarchical models, the musician-sequence interactions were significant [*F*_(28)_ = 3.58, *p* = 0.006, partial η^2^ = 0.28, Figure 5]. The TP of [0, −1, −2] with [1, 0.5], [0, 4,7] with [1, 0.5], and [0, −3, −2] with [1, 1.5] was significantly higher in W. J. Evans than M. Tyner (*p* = 0.031, *p* = 0.038, and *p* = 0.023, respectively). The TP of [0, 4, 7] with [1,1] was significantly higher in W. J. Evans than H. J. Hancock and M. Tyner (*p* < 0.001). The TP of [0, 7, 0] with [1, 1] was significantly higher in H. J. Hancock than M. Tyner (*p* = 0.029). The TP of [0, 4, 2] with [1, 2] was significantly higher in M. Tyner than W. J. Evans (*p* = 0.005) and H. J. Hancock (*p* = 0.007). The TP of [0, 2, 0] with [1, 3] was significantly higher in H. J. Hancock than W. J. Evans (*p* = 0.043). In the third-order hierarchical models, the musician-sequence interactions were significant [*F*_(28)_ = 4.91, *p* < 0.001, partial η^2^ = 0.35, Tables 3A,B and Figure 5]. The TP of [0, −1, −2, −3] with [1,0.5,0.5] was significantly higher in W. J. Evans than H. J. Hancock (*p* = 0.036) and M. Tyner (*p* = 0.007). The TP of [0, −2, −5, −7] with [1, 1, 1] was significantly lower in W. J. Evans than H. J. Hancock (*p* = 0.042). The TP of [0, −2, 2, 0] with [1, 1, 1], and [0, 5, 3, 0] with [1, 1, 1] was significantly higher in M. Tyner than W. J. Evans (*p* = 0.039 and *p* = 0.004, respectively). The TP of [0, −4, 3, 0] with [1, 1, 1] was significantly higher in M. Tyner than W. J. Evans (*p* = 0.031) and H. J. Hancock (*p* = 0.013). The TP of [0, 1, 5, 8] with [1, 1, 1] was significantly higher in W. J. Evans than H. J. Hancock (*p* = 0.011) and M. Tyner (*p* < 0.001). The TP of [0, 2, 4, 5] with [1, 1, 1] was significantly lower in M. Tyner than W. J. Evans (*p* < 0.001) and H. J. Hancock (*p* = 0.041). The TP of [0, 3, 0, 1] with [1, 1, 1] was significantly higher in W. J. Evans than H. J. Hancock (*p* = 0.027) and M. Tyner (*p* = 0.001). The TP of [0, 7, 4, 5] with [1, 1, 1] was significantly higher in W. J. Evans than H. J. Hancock (*p* = 0.027) and M. Tyner (*p* = 0.001). In the forth-order hierarchical models, the musician-sequence interactions were significant [*F*_(28)_ = 6.90, *p* < 0.001, partial η^2^ = 0.43, Tables 3A,B and Figure 5]. The TP of [0, −2, −3, −5, −6] with [1, 1, 1, 1], and [0, 1, 5, 8, 12] with [1, 1, 1, 1] was significantly lower in M. Tyner than W. J. Evans (all: *p* = 0.002). The TP of [0, −2, −4, 0, −2] with [1, 1, 1, 1], [0, −3, −7, −5, −3] with [1, 1, 1, 1], and [0, −3, 2, −1,−5] with [1, 1, 1, 1] was significantly higher in M. Tyner than W. J. Evans and H. J. Hancock (*p* = 0.008, *p* = 0.001, and *p* = 0.014, respectively). The TP of [0, −3, −2, 2, 5] with [1, 1, 1, 1] was significantly higher in W. J. Evans than H. J. Hancock (*p* = 0.009) and M. Tyner (*p* = 0.002). The TP of [0, −3, −2, 2, 5] with [1, 1, 1, 1] was significantly higher in W. J. Evans than H. J. Hancock and M. Tyner (all: *p* < 0.001). The TP of [0, −3, −5, −7, −5] with [1, 1, 1, 1] was significantly higher in M. Tyner than W. J. Evans (*p* = 0.017). The TP of [0, −3, −2, 2, 5] with [1, 1, 1, 1] was significantly higher in W. J. Evans than H. J. Hancock (*p* = 0.002) and M. Tyner (*p* < 0.001). The TP of [0, −3, −2, 2, 5] with [1, 1, 1, 1] was significantly higher in H. J. Hancock than M. Tyner (*p* = 0.035). In the fifth-order hierarchical models, the musician-sequence interactions were significant [*F*_(28)_ = 6.38, *p* < 0.001, partial η^2^ = 0.42, Tables 3A,B and Figure 5]. The TP of [0, −2, −3, −4, −5, −6] with [1, 1, 1, 1, 1], and [0, 1, 3, 5, 6, 8] with [1, 1, 1, 1, 1] was significantly lower in M. Tyner than H. J. Hancock (*p* = 0.022 and *p* = 0.035, respectively). The TP of [0, −2, −4, 0, −2, −4] with [1, 1, 1, 1, 1], and [0, −2, −5, 0, −2, −5] with [1, 1, 1, 1, 1] was significantly higher in M. Tyner than W. J. Evans and H. J. Hancock (all: *p* = 0.014). The TP of [0, −3, −2, 2, 5, 9] with [1, 1, 1, 1, 1], [0, 1, 3, 4, 6, 7] with [1, 1, 1, 1, 1], and [0,3,0,1,5,8] with [1, 1, 1, 1, 1] was significantly higher in Evans than H. J. Hancock and M. Tyner (all: *p* < 0.001). In the sixth-order hierarchical models, the musician-sequence interactions were significant [*F*_(28)_ = 4.20, *p* < 0.001, partial η^2^ = 0.32, Tables 3A,B and Figure 4]. The TP of [0, −2, −4, −7, −2, −4, −7] with [1, 1, 1, 1, 1, 1] was significantly higher in M. Tyner than W. J. Evans and H. J. Hancock (all: *p* = 0.014). The TP of [0, 3, 0, 1, 5, 8, 12] with [1, 1, 1, 1, 1, 1] was significantly higher in W. J. Evans than H. J. Hancock and M. Tyner (all: *p* = 0.001).

**Table 3:** The difference in TPs among pitch sequences with rhythms in each musician.

**Order**	**Sequence A**	**Sequence B**	**A-B**	**SE**	***p*-value**
**A. WJ. EVANS**					
3rd	0, −1, −2, −3 with 1, 0.5, 0.5	0, −2, 2, 0 with 1, 1, 1	1.461	0.333	0.038
	0, 1, 5, 8 with 1, 1, 1	0, −2, −5, −7 with 1, 1, 1	1.56	0.24	<0.001
		0, −4, 3, 0 with 1, 1, 1	1.569	0.356	0.035
		0, 1, 3, 5 with 1, 1, 1	1.318	0.295	0.032
4th	0, −2, −3, −5, −6 with 1, 1, 1, 1	0, −3, −7, −5, −3 with 1, 1, 1, 1	1.461	0.338	0.043
		0, −4, −2, 0, 1 with 1, 1, 1, 1	1.461	0.332	0.037
		0, 1, 3, 2, 1 with 1, 1, 1, 1	1.461	0.305	0.015
	0, −3, −2,0,1 with 1, 1, 1, 1	0, 1, 3, 2, 1 with 1, 1, 1, 1	1.362	0.301	0.027
	0,−3,−2,2,5 with 1, 1, 1, 1	0, −2, −4, 0, −2 with 1, 1, 1, 1	1.774	0.314	0.002
		0, −3, −5, −7, −5 with 1, 1, 1, 1	1.774	0.335	0.005
		0, −3, −7, −5, −3 with 1, 1, 1, 1	1.774	0.292	0.001
		0, −3, 2, −1, −5 with 1, 1, 1, 1	1.774	0.301	0.001
		0, −4, −2, 0, 1 with 1, 1, 1, 1	1.774	0.327	0.004
		0, 1, 3, 2, 1 with 1, 1, 1, 1	1.774	0.314	0.002
		0, 2, 3, 5, 7 with 1, 1, 1, 1	1.376	0.297	0.022
	0, 1, 5, 8,12 with 1, 1, 1, 1	0, −2, −4, 0, −2 with 1, 1, 1, 1	1.664	0.38	0.038
		0, −3, −7, −5, −3 with 1, 1, 1, 1	1.664	0.361	0.023
		0,−3, 2, −1, −5 with 1, 1, 1, 1	1.664	0.369	0.028
		0, −4, −2, 0, 1 with 1, 1, 1, 1	1.664	0.376	0.034
		0, 1, 3, 2, 1 with 1, 1, 1, 1	1.664	0.365	0.026
	0, 2, 3, 5, 6 with 1, 1, 1, 1	0, −3, −7, −5, −3 with 1, 1, 1, 1	1.328	0.289	0.024
		0, −3, 2, −1, −5 with 1, 1, 1, 1	1.328	0.299	0.033
		0, −4, −2, 0, 1 with 1, 1, 1, 1	1.328	0.31	0.047
5th	0, −3, −2, 2, 5, 9 with 1, 1, 1, 1, 1	0, −2, −3, −4, −5, −6 with 1, 1, 1, 1, 1	1.304	0.291	0.031
		0, −2, −4, 0, −2, −4 with 1, 1, 1, 1, 1	1.461	0.299	0.013
		0, −2, −5, 0, −2, −5 with 1, 1, 1, 1, 1	1.461	0.299	0.013
		0,−3,−2,0,−1,−2 with 1, 1, 1, 1, 1	1.461	0.312	0.02
		0, −3, −5, −7, −3, −5 with 1, 1, 1, 1, 1	1.461	0.312	0.02
		0, −3, −7, −5, −3, −7 with 1, 1, 1, 1, 1	1.461	0.312	0.02
		0, −4, 3, 0, −2, −4 with 1, 1, 1, 1, 1	1.461	0.312	0.02
	0, 1, 3, 4, 6, 7 with 1, 1, 1, 1, 1	0, −2, −3, −4, −5, −6 with 1, 1, 1, 1, 1	1.435	0.29	0.011
		0, −2, −4, 0, −2, −4 with 1, 1, 1, 1, 1	1.591	0.294	0.004
		0, −2, −5, 0, −2, −5 with 1, 1, 1, 1, 1	1.591	0.294	0.004
		0,−3,−2,0,−1,−2 with 1, 1, 1, 1, 1	1.591	0.308	0.007
		0, −3, −5, −7, −3, −5 with 1, 1, 1, 1, 1	1.591	0.308	0.007
		0, −3, −7, −5, −3, −7 with 1, 1, 1, 1, 1	1.591	0.308	0.007
		0, −4, 3, 0, −2, −4 with 1, 1, 1, 1, 1	1.591	0.308	0.007
	0, 3, 0, 1, 5, 8 with 1, 1, 1, 1, 1	0, −2, −3, −4, −5, −6 with 1, 1, 1, 1, 1	1.413	0.331	0.048
		0, −2, −4, 0, −2, −4 with 1, 1, 1, 1, 1	1.569	0.335	0.019
		0, −2, −5, 0, −2, −5 with 1, 1, 1, 1, 1	1.569	0.335	0.019
		0, −3, −2, 0, −1, −2 with 1, 1, 1, 1, 1	1.569	0.347	0.028
		0, −3, −5, −7, −3, −5 with 1, 1, 1, 1, 1	1.569	0.347	0.028
		0, −3, −7, −5, −3, −7 with 1, 1, 1, 1, 1	1.569	0.347	0.028
		0, −4, 3, 0, −2, −4 with 1, 1, 1, 1, 1	1.569	0.347	0.028
6th	0, 3, 0, 1, 5, 8, 12 with 1, 1, 1, 1, 1, 1	0,−1, −3, −4, −5, −6, −7 with 1, 1, 1, 1, 1, 1	1.413	0.322	0.038
		0, −2, −4, −7, −2, −4, −7 with 1, 1, 1, 1, 1, 1	1.413	0.331	0.048
		0, −2, −4, 0, −2, −4,−7 with 1, 1, 1, 1, 1, 1	1.413	0.327	0.043
		0, −2, −5, −7,−3,−5,−7 with 1, 1, 1, 1, 1, 1	1.413	0.327	0.043
		0, −2, −5, 0, −2, −5,−8 with 1, 1, 1, 1, 1, 1	1.413	0.322	0.038
**B. M. TYNER**					
3rd	0, 3, 0, 1 with 1, 1, 1	0, 4, 2, 0 with 1, 1, 1	−1.558	0.322	0.014
4th	0, −3, −7, −5, −3 with 1, 1, 1, 1	0, −3, −2, 0, 1 with 1, 1, 1, 1	1.413	0.327	0.044
		0, −3, −2, 2, 5 with 1, 1, 1, 1	1.413	0.292	0.014
		0, 2, 3, 5, 6 with 1, 1, 1, 1	1.413	0.289	0.013

#### Rhythm sequence with pitches

**Figure 6:**
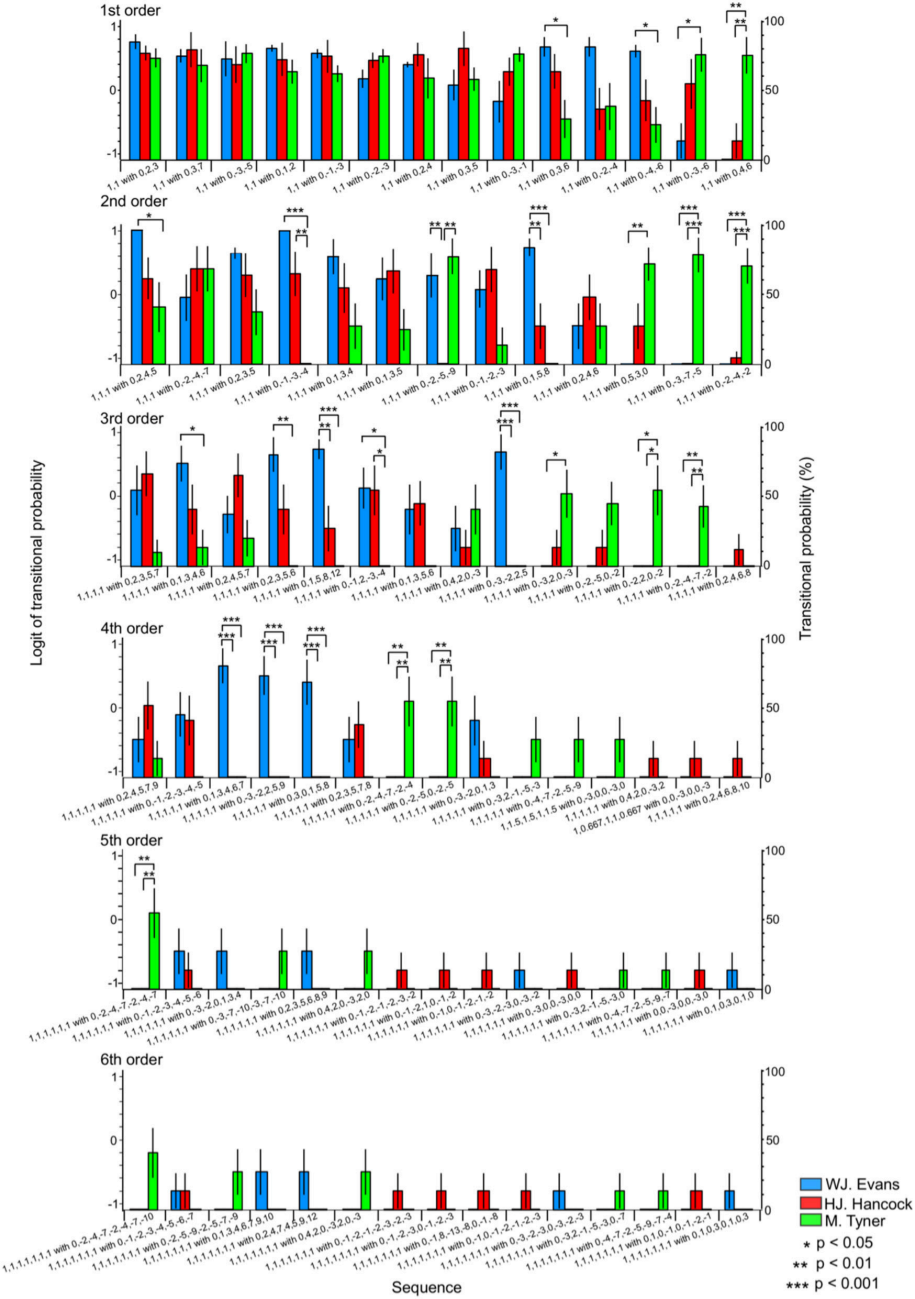
The difference in TPs among W.J. Evans (red), H.J. Hancock (blue), and M. Tyner (green) in rhythm sequence with pitches.

In the first-order hierarchical models, the main sequence effect were significant [*F*_(13, 234)_ = 4.45, *p* < 0.001, partial η^2^ = 0.20, Table 4]. The musician-sequence interactions were significant [*F*_(26)_ = 3.54, *p* < 0.001, partial η^2^ = 0.28, Figure 6 and Table 4]. The TP of [1,1] with [0, −3, −6] was significantly lower in W. J. Evans than M. Tyner (*p* = 0.037). The TP of [1, 1] with [0, −4, −6], and [1,1] with [0, 3, 6] was significantly higher in W. J. Evans than M. Tyner (all: *p* = 0.025). The TP of [1, 1] with [0, 4, 6] was significantly higher in M. Tyner than W. J. Evans (*p* = 0.001) and H. J. Hancock (*p* = 0.004). In the second-order hierarchical models, the musician-sequence interactions were significant [*F*_(24)_ = 5.53, *p* < 0.001, partial η^2^ = 0.42, Figure 6 and Table 4]. The TP of [1, 1, 1] with [0, −1, −3, −4] was significantly lower in M. Tyner than W. J. Evans (*p* < 0.001) and H. J. Hancock (*p* = 0.001). The TP of [1, 1, 1] with [0, −2, −4, −2], [1, 1, 1] with [0, −3, −7, −5] was significantly higher in M. Tyner than W. J. Evans and H. J. Hancock (*p* < 0.001). The TP of [1, 1, 1] with [0, −1, −3, −4] was significantly lower in H. J. Hancock than W. J. Evans (*p* = 0.007) and M. Tyner (*p* = 0.001). The TP of [1, 1, 1] with [0, −2, −4, −2], and [1, 1, 1] with [0, −3, −7, −5] was significantly higher in W. J. Evans than H. J. Hancock (*p* = 0.005) and M. Tyner (*p* < 0.001). The TP of [1, 1, 1] with [0, 2, 4, 5] was significantly lower in M. Tyner than W. J. Evans (*p* = 0.048). The TP of [1, 1, 1] with [0, 5, 3, 0] was significantly higher in M. Tyner than W. J. Evans (*p* = 0.002). In the third-order hierarchical models, the main sequence effect were significant [*F*_(5.05, 90.90)_ = 2.91, *p* = 0.017, partial η^2^ = 0.14, Table 4]. The musician-sequence interactions were significant [*F*_(26)_ = 5.88, *p* < 0.001, partial η^2^ = 0.40, Figure 6 and Table 4]. The TP of [1, 1, 1, 1] with [0, −1, −2, −3, −4] was significantly lower in M. Tyner than W. J. Evans (*p* = 0.040) and H. J. Hancock (*p* = 0.046). The TP of [1, 1, 1, 1] with [0, −2, −4, −7, −2], [1, 1, 1, 1] with [0, −2, 2, 0, −2] were significantly higher in M. Tyner than W. J. Evans (*p* = 0.008 and *p* = 0.015, respectively) and H. J. Hancock (*p* = 0.008 and *p* = 0.015, respectively). The TP of [1, 1, 1, 1] with [0, −3, −2, 2, 5] was significantly higher in W. J. Evans than H. J. Hancock and M. Tyner (all: *p* = 0.001). The TP of [1, 1, 1, 1] with [0, −3, 2, 0, −3] was significantly lower in W. J. Evans than M. Tyner (*p* = 0.038). The TP of [1, 1, 1, 1] with [0, 1, 3, 4, 6], and [1, 1, 1, 1] with [0, 2, 3, 5, 6] was significantly higher in W. J. Evans than M. Tyner (*p* = 0.046 and *p* = 0.002, respectively). The TP of [1, 1, 1, 1] with [0, 1, 5, 8, 12] was significantly higher in W. J. Evans than H. J. Hancock (*p* = 0.006) and M. Tyner (*p* < 0.001). In the forth-order hierarchical models, musician-sequence interactions were significant [*F*_(28)_ = 5.58, *p* < 0.001, partial η^2^ = 0.38, Figure 6 and Table 4]. The TP of [1, 1, 1, 1, 1] with [0, −2, −4, −7, −2, −4], and [1, 1, 1, 1, 1] with [0, −2, −5, 0, −2, −5] were significantly higher in M. Tyner than W. J. Evans and H. J. Hancock (all: *p* = 0.008). The TP of [1, 1, 1, 1, 1] with [0, −3, −2, 2, 5, 9], [1, 1, 1, 1, 1] with [0, 1, 3, 4, 6, 7], and [1, 1, 1, 1, 1] with [0, 3, 0, 1, 5, 8], were significantly higher in W. J. Evans than H. J. Hancock and M. Tyner (all: *p* < 0.001). In the fifth-order hierarchical models, the musician-sequence interactions were significant [*F*_(28)_ = 2.31, *p* < 0.001, partial η^2^ = 0.21, Figure 6 and Table 4]. The TP of [1, 1, 1, 1, 1, 1] with [0, −2, −4, −7, −2, −4, −7] was significantly higher in M. Tyner than W. J. Evans and H. J. Hancock (all: *p* = 0.008).

**Table 4:** The difference in TPs among rhythm sequences with pitches in each musician.

**Order**	**Sequence A**	**Sequence B**	**A-B**	**SE**	***p*-value**
**A. GENERAL**					
1st	1, 1 with 0, 4, 6	1, 1 with 0, −1, −3	−0.948	0.185	0.006
		1, 1 with 0, −2, −3	−0.883	0.177	0.009
		1, 1 with 0, 1, 2	−0.971	0.16	0.001
		1, 1 with 0, 2, 3	−1.114	0.176	0.001
		1, 1 with 0, 3, 7	−1.015	0.212	0.013
3rd	1, 1, 1, 1 with 0, 1, 3, 4, 6	1, 1, 1, 1 with 0, 2, 4, 6, 8	0.891	0.2	0.028
**B. WJ. EVANS**					
1st	1, 1 with 0, 4, 6	1, 1 with 0, −1, −3	−1.752	0.32	0.003
		1, 1 with 0, −2, −3	−1.337	0.307	0.035
		1, 1 with 0, −2, −4	−1.863	0.429	0.036
		1, 1 with 0, −4, −6	−1.791	0.331	0.004
		1, 1 with 0, 1, 2	−1.84	0.277	<0.001
		1, 1 with 0, 2, 3	−1.945	0.305	<0.001
		1, 1 with 0, 3, 6	−1.863	0.338	0.003
		1, 1 with 0, 3, 7	−1.716	0.367	0.017
2nd	1, 1, 1 with 0, −1, −3,−4	1, 1, 1 with 0, −2, −4,−2	2.197	0.327	<0.001
		1, 1, 1 with 0, −3, −7, −5	2.197	0.289	<0.001
		1, 1, 1 with 0, 2, 4, 6	1.569	0.359	0.028
		1, 1, 1 with 0,5,3,0	2.197	0.489	0.022
	1, 1, 1 with 0, −2, −4,−2	1, 1, 1 with 0,1,5,8	−1.924	0.329	0.001
		1, 1, 1 with 0, 2, 3,5	−1.83	0.387	0.013
	1, 1, 1 with 0, −3, −7, −5	1, 1, 1 with 0, −2, −5, −9	−1.461	0.329	0.025
		1, 1, 1 with 0,1,5,8	−1.924	0.308	0.001
	1, 1, 1 with 0,1,5,8	1, 1, 1 with 0,5,3,0	1.924	0.437	0.027
	1, 1, 1 with 0,2,4,5	1, 1, 1 with 0, −2, −4,−2	2.197	0.413	0.004
		1, 1, 1 with 0, −3, −7, −5	2.197	0.423	0.005
		1, 1, 1 with 0, 2, 4, 6	1.569	0.356	0.026
3rd	1, 1, 1, 1 with 0, −3, −2, 2, 5	1, 1, 1, 1 with 0, −2, −4,−7,−2	1.883	0.314	0.001
		1, 1, 1, 1 with 0, −2, −5, 0, −2	1.883	0.339	0.003
		1, 1, 1, 1 with 0, −2, 2, 0, −2	1.883	0.285	<0.001
		1, 1, 1, 1 with 0, −3, 2, 0, −3	1.883	0.356	0.004
		1, 1, 1, 1 with 0, 2, 4, 6, 8	1.883	0.242	<0.001
	1, 1, 1, 1 with 0, 1, 3, 4, 6	1, 1, 1, 1 with 0, −2, 2, 0, −2	1.696	0.373	0.022
		1, 1, 1, 1 with 0, 2, 4, 6, 8	1.696	0.346	0.01
	1, 1, 1, 1 with 0, 1, 5, 8, 12	1, 1, 1, 1 with 0, −2, −4,−7,−2	1.931	0.362	0.004
		1, 1, 1, 1 with 0, −2, −5, 0, −2	1.931	0.411	0.016
		1, 1, 1, 1 with 0, −2, 2, 0, −2	1.931	0.336	0.002
		1, 1, 1, 1 with 0, −3, 2, 0, −3	1.931	0.425	0.023
		1, 1, 1, 1 with 0, 2, 4, 6, 8	1.931	0.332	0.001
	1, 1, 1, 1 with 0, 2, 3, 5, 6	1, 1, 1, 1 with 0, −2, −4, −7, −2	1.844	0.404	0.022
		1, 1, 1, 1 with 0, −2, 2, 0, −2	1.844	0.382	0.012
		1, 1, 1, 1 with 0, 2, 4, 6, 8	1.844	0.391	0.016
4th	1, 1, 1, 1, 1 with 0, −3, −2, 2, 5,9	1, 1, 1, 1, 1 with 0, −2, −4, −7, −2, −4	1.678	0.325	0.007
		1, 1, 1, 1, 1 with 0, −2, −5, 0, −2,−5	1.678	0.325	0.007
		1, 1, 1, 1, 1 with 0,−3,0,0,−3,0,−1, 1.5,1.5,1, 1.5	1.678	0.269	0.001
		1, 1, 1, 1, 1 with 0, −3,2, −1, −5, −3	1.678	0.307	0.004
		1, 1, 1, 1, 1 with 0, −4, −7, −2, −5, −9	1.678	0.307	0.004
		1, 1, 1, 1, 1 with 0, 0, −3, 0, 0, −3, −1, 0.667, 1, 1, 0.667	1.678	0.269	0.001
		1, 1, 1, 1, 1 with 0, 2, 4, 6, 8,10	1.678	0.269	0.001
		1, 1, 1, 1, 1 with 0, 4, 2, 0, −3, 2	1.678	0.307	0.004
	1, 1, 1, 1, 1 with 0, 1, 3, 4, 6, 7	1, 1, 1, 1, 1 with 0, −2, −4,−7,−2,−4	1.839	0.312	0.001
		1, 1, 1, 1, 1 with 0, −2, −5, 0, −2, −5	1.839	0.312	0.001
		1, 1, 1, 1, 1 with 0, −3, 0, 0, −3, 0, −1, 1.5, 1.5, 1, 1.5	1.839	0.255	<0.001
		1, 1, 1, 1, 1 with 0, −3,2, −1, −5, −3	1.839	0.294	0.001
		1, 1, 1, 1, 1 with 0, −4, −7, −2, −5, −9	1.839	0.294	0.001
		1, 1, 1, 1, 1 with 0, 0, −3, 0, 0, −3, −1, 0.667, 1, 1, 0.667	1.839	0.255	<0.001
		1, 1, 1, 1, 1 with 0, 2, 4, 6, 8,10	1.839	0.255	<0.001
		1, 1, 1, 1, 1 with 0, 4, 2, 0, −3, 2	1.839	0.294	0.001
	1, 1, 1, 1, 1 with 0, 3, 0, 1, 5, 8	1, 1, 1, 1, 1 with 0, −2, −4, −7, −2, −4	1.569	0.347	0.028
		1, 1, 1, 1, 1 with 0, −2, −5, 0, −2, −5	1.569	0.347	0.028
		1, 1, 1, 1, 1 with 0, −3, 0, 0, −3, 0, −1, 1.5, 1.5, 1, 1.5	1.569	0.296	0.005
		1, 1, 1, 1, 1 with 0, −3, 2, −1, −5, −3	1.569	0.331	0.017
		1, 1, 1, 1, 1 with 0, −4, −7, −2, −5, −9	1.569	0.331	0.017
		1, 1, 1, 1, 1 with 0, 0, −3, 0, 0, −3, −1, 0.667, 1, 1, 0.667	1.569	0.296	0.005
		1, 1, 1, 1, 1 with 0, 2, 4, 6, 8,10	1.569	0.296	0.005
		1, 1, 1, 1, 1 with 0, 4, 2, 0, −3, 2	1.569	0.331	0.017
**C. H. J. HANCOCK**					
1st	1, 1 with 0, 4, 6	1, 1 with 0, −1, −3	−1.394	0.32	0.035
		1, 1 with 0, −2, −3	−1.327	0.307	0.038
		1, 1 with 0, 1, 2	−1.343	0.277	0.012
		1, 1 with 0, 2, 3	−1.445	0.305	0.015
		1, 1 with 0, 3, 5	−1.519	0.355	0.041
2nd	1, 1, 1 with 0, −1, −3,−4	1, 1, 1 with 0, −2, −4, −2	1.377	0.327	0.041
		1, 1, 1 with 0, −3, −7, −5	1.485	0.289	0.005
3rd	1, 1, 1, 1 with 0, −3, −2, 2, 5	1, 1, 1, 1 with 0, 2, 3,5,7	−1.525	0.353	0.038
		1, 1, 1, 1 with 0, 2, 4, 5, 7	−1.501	0.357	0.048
**D. M. TYNER**					
2nd	1, 1, 1 with 0, −1, −3,−4	1, 1, 1 with 0, −2, −4,−2	−1.618	0.327	0.008
		1, 1, 1 with 0, −2, −5, −9	−1.774	0.368	0.011
		1, 1, 1 with 0, −3, −7, −5	−1.799	0.289	0.001
	1, 1, 1 with 0,1,5,8	1, 1, 1 with 0, −2, −4,−2	−1.618	0.329	0.009
		1, 1, 1 with 0, −2, −5, −9	−1.774	0.342	0.005
		1, 1, 1 with 0, −3, −7, −5	−1.799	0.308	0.001
3rd	1, 1, 1, 1 with 0, −2, −5, 0, −2	1, 1, 1, 1 with 0, 2, 4, 6, 8	1.038	0.223	0.018

### Regression analysis

#### Pitch sequence without rhythms

Results were shown in Table 5A. In W. J. Evans, no significant regression equation was detected in the first-, second-, forth-, and sixth-order hierarchical models. In the third-order hierarchical model, a significant regression equation was found [*F*_(2, 4)_ = 16.19, *p* = 0.012], with an adjusted *R*^2^ of 0.84. The predicted chronological order is equal to 9.43–17.98 (transition of [0, −3, −7, −5]) −7.23 (transition of [0, 2, 4, 5]). The TPs of [0, 2, 4, 5] and [0, −3, −7, −5] gradually decreased consistently with the ascending chronological order ([0, −3, −7, −5] *p* = 0.007, [0, 2, 4, 5] *p* = 0.031). In the fifth-order hierarchical model, a significant regression equation was found [*F*_(1, 5)_ = 14.74, *p* = 0.012], with an adjusted *R*^2^ of 0.70. The predicted chronological order is equal to 5.33–9.31 (transition of [0, 2, 3, 5, 7, 8]). The TPs of [0, 2, 3, 5, 7, 8] gradually decreased consistently with the ascending chronological order (*p* = 0.012). In H. J. Hancock, no significant regression equation was detected in all of the hierarchical models. In M. Tyner, no significant regression equation was detected in the first-, third-, forth-, fifth-, and sixth-order hierarchical models. Only in the second-order hierarchical model, a significant regression equation was found [*F*_(2, 4)_ = 31.04, *p* = 0.004], with an adjusted *R*^2^ of 0.91. The predicted chronological order is equal to −3.68 + 28.30 (transition of [0, −2, −5]) + 10.59 (transition of [0, 2, 0]). The TPs of [0, −2, −5] and [0, 2, 0] gradually increased consistently with the ascending chronological order ([0, −2, −5] *p* = 0.003, [0, 2, 0] *p* = 0.038). These TPs were significant predictors of the chronological order.

**Table 5:** Regression analyses based on the stepwise method.

			**Model 1**					**Model 2**				
		**Variable**	***B***	**SE B**	***β***	**VIF**	***CI***	***B***	**SE B**	***β***	**VIF**	***CI***
**A. PITCH TRANSITION**												
W. J. Evans	Third	0, −3, −7, −5	−16.36	5.99	−0.77^*^	1.00	2.36	−17.98	3.54	−0.85^**^	1.02	2.45
		0, 2, 4, 5						−7.22	2.22	−0.55^*^	1.02	8.40
		*R*^2^	0.52					0.84				
		*F*	7.46^*^					16.19^*^				
	Fifith	0, 2, 3, 5, 7, 8	−5.58	1.45	−0.86^*^	1.00	2.03					
		*R*^2^	0.70									
		*F*	14.74^*^									
M. Tyner	Second	0, −2, −5	31.21	7.67	0.88^*^	1.00	7.34	28.30	4.47	0.80^**^	1.04	5.18
		0,2,0						10.59	3.15	0.42^*^	1.04	8.96
		*R*^2^	0.72					0.91				
		*F*	16.56^*^					31.04^**^				
**B. RHYTHM TRANSITION**												
W. J. Evans	Second	1, 2, 2	17.75	4.33	0.88^**^	1.00	6.19					
		*R*^2^	0.73									
		*F*	16.85^**^									
H. J. Hancock	First	1, 0.333	−506.2	129.84	−0.87^*^	1.00	10.04	−471.7	76.01	−0.81^**^	1.02	2.83
		1, 1.5						−40.48	12.27	−0.43^*^	1.02	11.62
		*R*^2^	0.70					0.90				
		*F*	15.20^*^					28.05^**^				
M. Tyner	Forth	1, 2, 1, 2, 1	5.37	1.78	0.80^*^	1.00	4.20					
		*R*^2^	0.57									
		*F*	9.08^*^									
**C. PITCH TRANSITION WITH RHYTHM**												
H. J. Hancock	Second	0,4,2 with 1,2	10.50	3.64	0.79^*^	1.00	1.82					
		*R*^2^	0.55									
		*F*	8.33^*^									
M. Tyner	Third	0, 0, 0, 0 with 1, 1, 1	−3.72	1.03	−0.85^*^	1.00	2.16					
		*R*^2^	0.67									
		*F*	12.99^*^									
	Forth	0, −3, 2, −1, −5 with 1, 1, 1, 1	−3.33	1.23	−0.77^*^	1.00	2.55					
		*R*^2^	0.51									
		*F*	7.35^*^									
**D. RHYTHM TRANSITION WITH PITCH**												
W. J. Evans	Second	1, 1, 1 with 0, 2, 4, 6	−3.50	1.21	−0.79^*^	1.00	1.82	−4.72	0.90	−1.07^**^	1.30	2.20
		1, 1, 1 with 0, −2, −5, −9						2.61	0.93	0.57^*^	1.30	4.09
		*R*^2^	0.55					0.81				
		F	8.33^*^					13.80^*^				
	Forth	1, 1, 1, 1, 1 with 0, −3, −2, 0, 1, 3	−629.6	149.6	−0.88^**^	1.00	1.00					
		*R*^2^	0.74									
		F	17.72^**^									
	Fifth	1, 1, 1, 1, 1, 1 with 0, −3, −2, 0, 1, 3,4	−3.50	1.21	−0.79^*^	1.00	1.82					
		*R*^2^	0.55									
		*F*	8.33^*^									
H. J. Hancock	First	1, 1 with 0, −2, −3	−11.40	2.94	−0.87^*^	1.00	10.07					
		*R*^2^	0.70									
		*F*	15.06^*^									

* *p < 0.05*,

** *p < 0.01. SE, standard error; VIF, variance inflation factor**;** CI, condition index*.

#### Rhythm sequence without pitches

Results were shown in Table 5B. In W. J. Evans, no significant regression equation was detected in the first-, third-, forth-, fifth-, and sixth-order hierarchical models. In the second-order hierarchical model, a significant regression equation was found [*F*_(1, 5)_ = 16.85, *p* = 0.009], with an adjusted *R*^2^ of 0.73. The predicted chronological order is equal to −1.29 + 17.75 (transition of [1, 2, 2]). The TPs of [1, 2, 2] gradually increased consistently with the ascending chronological order (*p* = 0.009). In H. J. Hancock, no significant regression equation was detected in the second-, third-, forth-, fifth-, and sixth-order hierarchical models. In the first-order hierarchical model, a significant regression equation was found [*F*_(3, 3)_ = 82.70, *p* = 0.002], with an adjusted *R*^2^ of 0.98. The predicted chronological order is equal to 12.73–583.67 (transition of [1, 0.333])−79.86 (transition of [1, 1.5]) + 33.53 (transition of [1, 2]). The TPs of [1, 0.333] and [1, 1.5] gradually decreased and those of [1, 2] gradually increased consistently with the ascending chronological order (*p* = 0.001, *p* = 0.007, and *p* = 0.034, respectively). In M. Tyner, no significant regression equation was detected in the first-, second-, third-, fifth-, and sixth-order hierarchical models. In the forth-order hierarchical model, a significant regression equation was found [*F*_(1, 5)_ = 9.08, *p* = 0.030], with an adjusted *R*^2^ of 0.57. The predicted chronological order is equal to 0.82 + 5.37 (transition of [1, 2, 1, 2, 1]). The TPs of [1, 2, 1, 2, 1] gradually increased consistently with the ascending chronological order (*p* = 0.030). These TPs were significant predictors of the chronological order.

#### Pitch sequence with rhythms

Results were shown in Table 5C. In W. J. Evans, no significant regression equation was detected in all of the hierarchical models. In H. J. Hancock, no significant regression equation was detected in the first-, third-, forth-, fifth-, and sixth-order hierarchical models. In the second-order hierarchical model, a significant regression equation was found [*F*_(1, 5)_ = 8.33, *p* = 0.034], with an adjusted *R*^2^ of 0.55. The predicted chronological order is equal to 3.0 + 10.50 (transition of [0, 4, 2] with [1, 2]). The TPs of [0, 4, 2] with [1, 2] gradually increased consistently with the ascending chronological order (*p* = 0.034). In M. Tyner, no significant regression equation was detected in the first-, second-, fifth-, and sixth-order hierarchical models. In the third-order hierarchical model, a significant regression equation was found [*F*_(1, 5)_ = 12.99, *p* = 0.015], with an adjusted *R*^2^ of 0.67. The predicted chronological order is equal to 5.44–3.72 (transition of [0, 0, 0, 0] with [1, 1, 1]). The TPs of [0, 0, 0, 0] with [1, 1, 1] gradually decreased consistently with the ascending chronological order (*p* = 0.015). In the forth-order hierarchical model, a significant regression equation was found [*F*_(1, 5)_ = 7.35, *p* = 0.042], with an adjusted *R*^2^ of 0.51. The predicted chronological order is equal to 5.67–3.33 (transition of [0, −3, 2, −1, −5] with [1, 1, 1, 1]). The TPs of [0, −3, 2, −1, −5] with [1, 1, 1, 1] gradually decreased consistently with the ascending chronological order (*p* = 0.042). These TPs were significant predictors of the chronological order.

#### Rhythm sequence with pitches

Results were shown in Table 5D. In W. J. Evans, no significant regression equation was detected in the first-, third-, and sixth-order hierarchical models. In the second-order hierarchical model, a significant regression equation was found [*F*_(2, 4)_ = 13.80, *p* = 0.016], with an adjusted *R*^2^ of 0.81. The predicted chronological order is equal to 3.61–4.72 (transition of [1, 1, 1] with [0, 2, 4, 6]) + 2.61 (transition of [1, 1, 1] with [0, −2, −5, −9]). The TPs of [1, 1, 1] with [0, 2, 4, 6] gradually decreased (*p* = 0.006) and the TPs of [1, 1, 1] with [0, −2, −5, −9] gradually increased (*p* = 0.049) consistently with the ascending chronological order. In the forth-order hierarchical model, a significant regression equation was found [*F*_(1, 5)_ = 17.72, *p* = 0.008], with an adjusted *R*^2^ of 0.74. The predicted chronological order is equal to 5.33–629.65 (transition of [1, 1, 1, 1, 1] with [0, −3, −2, 0, 1, 3]). The TPs of [1, 1, 1, 1, 1] with [0, −3, −2, 0, 1, 3] gradually decreased consistently with the ascending chronological order (*p* = 0.008). In the fifth-order hierarchical model, a significant regression equation was found [*F*_(1, 5)_ = 8.33, *p* = 0.034], with an adjusted *R*^2^ of 0.55. The predicted chronological order is equal to 5.00–3.50 (transition of [1, 1, 1, 1, 1, 1] with [0, −3, −2, 0, 1, 3, 4]). The TPs of [1, 1, 1, 1, 1, 1] with [0, −3, −2, 0, 1, 3, 4] gradually decreased consistently with the ascending chronological order (*p* = 0.034). In H. J. Hancock, no significant regression equation was detected in the second-, third-, forth-, fifth-, and sixth-order hierarchical models. In the first-order hierarchical model, a significant regression equation was found [*F*_(1, 5)_ = 15.06, *p* = 0.012], with an adjusted *R*^2^ of 0.70. The predicted chronological order is equal to 12.64–11.40 (transition of [1, 1] with [0, −2, −3]). The TPs of [1, 1] with [0, −2, −3] gradually decreased consistently with the ascending chronological order (*p* = 0.012). These TPs were significant predictors of the chronological order. In M. Tyner, no significant regression equation was detected in all of the hierarchical models.

#### Time-course variation of entropy

Results were shown in Table 6. In the rhythm sequence with pitches in H. J. Hancock, significant regression equation was detected in the higher- but not lower-order hierarchical models. In the fifth-order hierarchical model, a significant regression equation was found [*F*_(1, 5)_ = 10.58, *p* = 0.023], with an adjusted *R*^2^ of 0.62. The predicted chronological order is equal to 5.73–193.34. The entropies of rhythm sequence with pitches gradually decreased (*p* = 0.023) consistently with the ascending chronological order. In the sixth-order hierarchical model, a significant regression equation was found [*F*_(1, 5)_ = 9.28, *p* = 0.029], with an adjusted *R*^2^ of 0.58. The predicted chronological order is equal to 5.67–272.31. The entropies of rhythm sequence with pitches gradually decreased (*p* = 0.029) consistently with the ascending chronological order. No significant regression equation was detected in W.J. Evans and M.Tyner.

**Table 6:** Time–course variation of entropy (rhythm sequence with entropies).

		**Model**				
**Hierarchy**	**Variable**	***B***	**SE B**	**β**	**VIF**	***CI***
Fiffth	Rhythm sequence with pitches in Hancock	−193.34	59.44	−0.82^*^	1.00	2.50
	*R*^2^	0.62				
	*F*	10.58^*^				
Sixth	Rhythm sequence with pitches in Hancock	−272.31	89.38	−0.81^*^	1.00	2.47
	*R*^2^	0.58				
	*F*	9.28^*^				

* *p < 0.05. SE, standard error; VIF, variance inflation factor; CI, condition index*.

## Discussion

### Interpretation of multi-order hierarchical models for implicit learning

In the context of implicit-learning models on information theory and predictive coding (Friston, 2005; Pearce and Wiggins, 2012; Rohrmeier and Rebuschat, 2012), the TP distribution sampled from musical improvisation based on n-order Markov models may refer to the characteristics of a composer's superficial-to-deep (i.e., n-order) implicit knowledge: a tone with high TP compared to a tone with a low TP may be one that a composer is more likely to predict and choose based on the latest n tones. The notion has been neurophysiologically demonstrated by our previous studies on predictive coding (Daikoku et al., 2017b). Using the various-order Markov stochastic models that unify temporal and spectral features in musical improvisation, the present study investigated the stochastic difference of temporal and spectral features among musicians, and clarified which information (pitch and rhythm) and depth (1st to 6th orders) represent the individualities of improvisational creativity and how they interact with each other.

### Hierarchy

The results of principal component analysis (PCA) suggested that the lower-order models represented general statistical characteristics shared among musicians, whereas higher-order models represented specific statistical characteristics that were unique to each musician (Figure 1). In the 1st-order models of any type of temporal and spectral sequences, and 2nd-order models of sequences other than pitch sequence with rhythm, component 1 showed general characteristics in improvisation. These results suggest that the individuality of improvisational creativity depends on the depth of implicit knowledge. This hypothesis could also be underpinned by ANOVA results. To understand the differences between TPs in each sequence among musicians, only the transitional patterns with first to fifth highest TPs from each musician, which showed higher predictabilities in each musician, were analyzed using an ANOVA. In lower-order models, universal sequences that are common among musicians could be detected. For example, in a 1st-order model of pitch sequence without rhythm (Figure 3, top), the extracted sequences of [0, 0], [0, −1], [0, 1], [0, −2], [0, 2], [0, −3], and [0, 3] correspond to repetition of the same tone, and semi-tone, whole-tone, and minor-third transitions. These sequences are frequently exploited in many types of music (e.g., Classical, Jazz), are easier to immediately play because of the small pitch intervals, and lead to a smooth melody. However, in the 6th-order model (Figure 3, bottom), the TPs for the sequences of [0, 3, 0, 1, 5, 8, 12] and [0, −2, −4, −7, −2, −4, −7] were different among musicians. Although the difference could also be detected even in the 1st-order model, higher-order models showed a larger difference of TPs among musicians, suggesting that individuality of musical prediction and production is larger with a deeper implicit knowledge. In summary, the results of the present study suggest that the individuality of improvisational creativity may be formed by deeper implicit knowledge, whereas lower-order implicit knowledge may be shared among musicians.

### Pitch and rhythm

In the pitch sequences with and without rhythms and the rhythm sequence with pitches (Figures 1A,C,D), W. J. Evans' and M. Tyner's components could be detected in any-order model. In a rhythm sequence without pitch (Figure 1B), however, no obvious difference among musicians could be detected. These results suggest that individuality of musical creativity is shaped by spectral, rather than temporal, implicit knowledge. However, the results also suggest that temporal knowledge at least contributes to formation of individuality; TP distribution of pitch sequences “with” rhythms, compared to those “without” rhythms, showed clear individuality among three musicians from a lower-order model (i.e., 4th-order model). Additionally, in two types of rhythm sequences without and with pitches (Figures 2B,D, respectively), TP distribution with, but not without, pitches showed individuality of improvisation. This suggests that temporal and spectral implicit knowledge interact with each other. The ANOVA results support these PCA findings. In lower-order models, the extent of the difference in TPs among musicians is larger for pitch sequences with rhythms (**Figure 7**) than for those without rhythm (Figure 3). Additionally, in two types of rhythm sequences without and with pitches (Figures 5, **7**, respectively), the extent of the TP difference among musicians is larger in rhythm sequences with, compared to without, pitches. Together, these results suggest that the individuality of improvisational creativity may essentially be formed by pitch, but not rhythm, implicit knowledge. However, implicit knowledge of rhythm may strengthen individuality.

### Time-course variation of implicit knowledge

In all types of spectro-temporal sequences of each hierarchy, time-course variation of TPs in some sequences could be detected. There were two types of time-course variations: TPs that gradually decrease, and those that gradually increase, consistent with the chronological order. Thus, implicit knowledge of pitch and rhythm could be shifted over a musician's life. However, the findings suggested that the time-course variations in TPs do not depend on hierarchy and spectro-temporal features, while the individuality among musicians may depend on these features. This suggests that the shifts in implicit knowledge may occur in each musician's lifetime, regardless of spectro-temporal features and the depth of knowledge. It may be interesting to investigate if the findings of gradual shifts in TPs reflect those of implicit knowledge via experience and training. Learning to play the piano enhances auditory-motor skills based on procedural knowledge (Norgaard, 2014), which corresponds to implicit knowledge (Clark and Squire, 1998; Ullman, 2001; Paradis, 2004; De Jong, 2005; Ellis, 2009; Müller et al., 2016). Thus, through experience and long-term training over the player's life, implicit knowledge that is tied to musical expression may shift (Daikoku et al., 2012). On the other hand, the time-course variations of the entropies, which represent uncertainly in music (Pearce and Wiggins, 2006), could be detected in higher-order hierarchy in one musician. Future study is needed to investigate the relationships of time-course variation between specific phrase and general uncertainty. In addition, the results of the present study cannot completely support the hypothesis because time-course variations among only seven pieces of music for each musician were investigated. Further research is needed to verify a larger number of music pieces in a musician's lifetime, and to examine behavioral and neurophysiological results.

### General discussion: informatics and neural aspects in musical creativity

In summary, the present study found three types of results on improvisational music and implicit knowledge: hierarchy, spectro-temporal features, and time-course variation. First, the lower-order TP distribution represented general characteristics shared among musicians, whereas higher-order TP distribution detected specific characteristics that were unique to each musician. Thus, the individuality of improvisational creativity might be formed by deeper (i.e., higher-order), but not superficial (i.e., lower-order), implicit knowledge. Second, the TP distribution with pitch information detected specific characteristics that were unique to each musician, whereas the TP distribution with only rhythm information could not detect differences among musicians. Thus, the individuality of improvisational creativity may essentially be formed by spectral (i.e., pitch), but not temporal (i.e., rhythm), implicit knowledge, whereas the rhythms may allow the individuality of pitches to strengthen. Third, TPs of some phrase were gradually decreased, and increased consistent with the chronological order for each musician, regardless of hierarchy and spectro-temporal feature in the TP distributions. Thus, time-course variation of implicit knowledge in pitches and rhythms may occur throughout a musician's lifetime regardless of the depth of knowledge. On the other hand, the time-course variations of the entropies, which represent uncertainly in music (Pearce and Wiggins, 2006), could be detected in higher-order hierarchy in one musician.

It is generally considered that musical expression in improvisation is mainly shaped by tacit knowledge (Delie‘ge et al., 1996; Koelsch et al., 2000; Delie‘ge, 2001; Bigand and Poulin-Charronnat, 2006; Ettlinger et al., 2011; Koelsch, 2011; Huron, 2012). Particularly, the expression of musical improvisation, compared to other types of musical composition in which a composer deliberates a composition scheme for a long time based on musical theory, forces musicians to continually predict each forthcoming tone, and immediately play the melody based on intuitive decision-making and auditory-motor planning, which are considered to tie in with procedural and implicit knowledge (Berry and Dienes, 1993; Reber, 1993; Clark and Squire, 1998; Ullman, 2001; Paradis, 2004; De Jong, 2005; Ellis, 2009; Norgaard, 2014; Müller et al., 2016; Perkovic and Orquin, 2017). Thus, the musical improvisation may be more strongly related to the implicit knowledge, compared to other types of music. Few studies have investigated the relationship between musical improvisation and implicit learning via computational model (Norgaard, 2014) and neural correlate (Adhikari et al., 2016; Lopata et al., 2017). In a series of my previous neurophysiological studies using Markov stochastic models and other studies on music, implicit learning of pitch, harmony, and dyad chord could be reflected in event-related responses (ERP/ERF) based on predictive coding (Daikoku et al., 2014, 2015, 2016, 2017a; Daikoku and Yumoto, 2017; Moldwin et al., 2017). Other studies also detected neural correlates to the motor control for auditory prediction and production when playing the piano (Bianco et al., 2016), and to improvisational creativity of music (Pinho et al., 2015; Adhikari et al., 2016; Lopata et al., 2017). These studies suggest that the mental representation of a musician's knowledge facilitates optimisation of motor actions (Daikoku et al., 2018) in the framework of information theory on brain function. The findings of the present study were based on relative but not absolute stochastic feature of music. Thus, the results in this study could support the previous neurophysiological and psychological studies that suggest that human's brain learn relative rather than absolute temporal and spectral (Daikoku et al., 2014, 2015) patterns.

The verification of computational models and the neural correlates have also been performed in previous studies (see review, Rohrmeier and Rebuschat, 2012). For example, the n-gram models calculate probability of sequential patterns by chopping them into short fragments (n-grams) up to a size of n. This model, which is frequently verified by neural approaches, is considered to correspond to chunking and word-segmentation processes in implicit learning (Saffran et al., 1996). The online perception and production of real-world dynamical music, however, is not the mere chopping of sequential patterns like word segmentation, but dynamical prediction to maintain an aesthetic melody with various temporal and spectral features, hierarchical structure, and harmony, which interact with each other (Lerdahl and Jackendoff, 1983; Hauser et al., 2002; Jackendoff and Lerdahl, 2006). Musical prediction and the representation constantly occurs with each state of sequences during learning and playing music. In addition, they are not restricted to a single stream of events or hierarchy but, rather, they interact with each parallel stream (Conklin and Witten, 1995; Pearce and Wiggins, 2012). Given the real-world phenomenon of music perception and prediction, various-order Markov models may be able to express dynamical and hierarchical creativity that occur in a musician's brain when they play music (Pearce and Wiggins, 2012), and to interdisciplinarily verify lower-to-deeper implicit knowledge and its representation using one experiment via neurophyisiological and informatics approaches. Using the models, however, future study is needed to also investigate other aspects of music such as harmony, non-adjacent dependency, and tree-structure nature of melody and harmony.

In conclusion, the present study suggested that the formation of individuality of musical creativity may depend on spectro-temporal features and hierarchy. The present study first provides the hierarchical implicit-learning model that unifies temporal and spectral features in musical improvisationa and creativity and that is interdisciplinarily verifiable using neurophysiological, behavioral, and information-thepretic approaches.

## Data availability

All relevant data are within the paper.

## Author contributions

The author confirms being the sole contributor of this work and has approved it for publication.

### Conflict of interest statement

The author declares that the research was conducted in the absence of any commercial or financial relationships that could be construed as a potential conflict of interest.
